# CREB-binding protein/P300 bromodomain inhibition reduces neutrophil accumulation and activates antitumor immunity in triple-negative breast cancer

**DOI:** 10.1172/jci.insight.182621

**Published:** 2024-09-17

**Authors:** Xueying Yuan, Xiaoxin Hao, Hilda L. Chan, Na Zhao, Diego A. Pedroza, Fengshuo Liu, Kang Le, Alex J. Smith, Sebastian J. Calderon, Nadia Lieu, Michael J. Soth, Philip Jones, Xiang H.F. Zhang, Jeffrey M. Rosen

**Affiliations:** 1 Department of Molecular and Cellular Biology and; 2 Lester and Sue Smith Breast Center, Baylor College of Medicine, Houston, Texas, USA.; 3 Institute for Applied Cancer Science (IACS), University of Texas MD Anderson Cancer Center, Houston, Texas, USA.

**Keywords:** Immunology, Oncology, Breast cancer, Cellular immune response, Epigenetics

## Abstract

Tumor-associated neutrophils (TANs) have been shown to promote immunosuppression and tumor progression, and a high TAN frequency predicts poor prognosis in triple-negative breast cancer (TNBC). Dysregulation of CREB-binding protein (CBP)/P300 function has been observed with multiple cancer types. The bromodomain (BRD) of CBP/P300 has been shown to regulate its activity. In this study, we found that IACS-70654, a selective CBP/P300 BRD inhibitor, reduced TANs and inhibited the growth of neutrophil-enriched TNBC models. In the bone marrow, CBP/P300 BRD inhibition reduced the tumor-driven abnormal differentiation and proliferation of neutrophil progenitors. Inhibition of CBP/P300 BRD also stimulated the immune response by inducing an IFN response and MHCI expression in tumor cells and increasing tumor-infiltrated cytotoxic T cells. Moreover, IACS-70654 improved the response of a neutrophil-enriched TNBC model to docetaxel and immune checkpoint blockade. This provides a rationale for combining a CBP/P300 BRD inhibitor with standard-of-care therapies in future clinical trials for neutrophil-enriched TNBC.

## Abstract 2

CREB binding protein/P300 bromodomain inhibition reduces tumor growth and systemic neutrophil accumulation while stimulating an antitumor immune response in triple-negative breast cancer.

## Graphical abstract

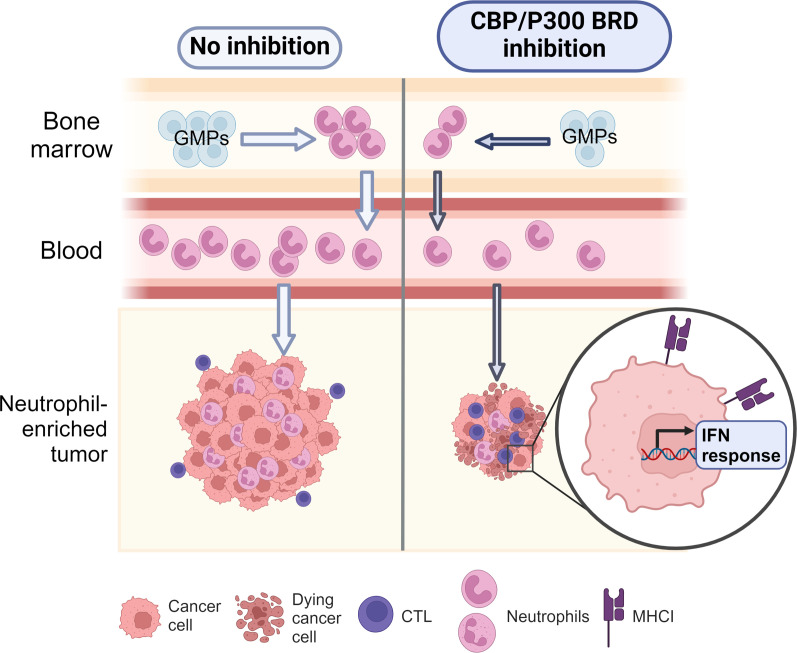

## Introduction

Triple-negative breast cancer (TNBC) is a biologically heterogeneous and clinically important breast cancer subtype defined by the lack of estrogen receptor, progesterone receptor, and human epidermal growth factor receptor 2 amplification (1). In the tumor immune microenvironment (TIME) of TNBC, tumor-associated myeloid cells, including tumor-associated neutrophils (TANs) and tumor-associated macrophages (TAMs), are the most abundant infiltrated immune cells (2, 3). Immunosuppressive TANs can be categorized as polymorphonuclear myeloid-derived suppressor cells (PMN-MDSCs), and their development and phenotype are distinct from normal neutrophils (4, 5). They contribute to tumor progression through T cell inhibition, promoting tumor proliferation and therapy resistance (3, 4, 6). TNBC exhibits heterogeneous frequencies of TANs, and high infiltration of TANs has been associated with poor prognosis (2, 7). Moreover, systemic changes such as the accumulation of blood neutrophils and overproduction of immature myeloid cells in the bone marrow (BM) have been observed in TNBC (7–10). TANs can also facilitate tumor metastasis by inducing invasion, migration, and epithelial-mesenchymal transition (EMT) (3, 6).

Epigenetic modifications have been demonstrated to reprogram the functions and accumulation of TANs (11, 12). Previously, we performed an organoid screen in search of epigenetic inhibitors that can reverse EMT, and IACS-70654, a selective CREB-binding protein (CBP)/P300 bromodomain (BRD) inhibitor, was identified as one of the top hits in this screen (13). CBP and P300 are transcriptional coactivators that are highly homologous, especially in sequences encoding histone acetyltransferase (HAT) domains and BRDs (14, 15). The HAT domain of CBP/P300 can acetylate histones and some transcription factors (e.g., p53) to regulate gene expression, and BRD can facilitate acetylation by the HAT domain (15–19). Functional dysregulation of CBP/P300 has been associated with tumorigenesis (16). It has been difficult to develop selective inhibitors of the HAT domains and progress them to the clinic, but inhibitors targeting BRDs have shown promising results (20). CBP/P300 BRD inhibition can reduce acetylation by CBP/P300, but BRD inhibition is not equivalent to HAT inhibition in magnitude and sites of reduced acetylation (17). The CBP/P300 BRD inhibitor CCS147 is in clinical trials for hematologic malignancies (21, 22). More importantly, inhibition of P300/CBP BRD was able to reduce the growth of the human TNBC xenograft model MDA-MB-231 X1.1 and reprogrammed the tumor-associated myeloid cells (23). However, the effects of CBP/P300 BRD inhibition on TNBC and the TIME have not been thoroughly investigated in immunocompetent models. Therefore, this study was designed to test the effects of CBP/P300 BRD inhibition on both tumor cells and the TIME of multiple immunocompetent mouse models of TNBC with heterogeneous TAN and TAM frequencies. We found that CBP/P300 BRD inhibition suppressed the growth of neutrophil-enriched TNBC models and reduced immunosuppression by decreasing TANs and inhibiting abnormal neutrophil production in BM. CBP/P300 BRD inhibition also exerted immune stimulation by inducing both an IFN and a T cell response.

## Results

### IACS-70654 inhibited the growth of neutrophil-enriched syngeneic mammary tumor models.

**Figure 1:**
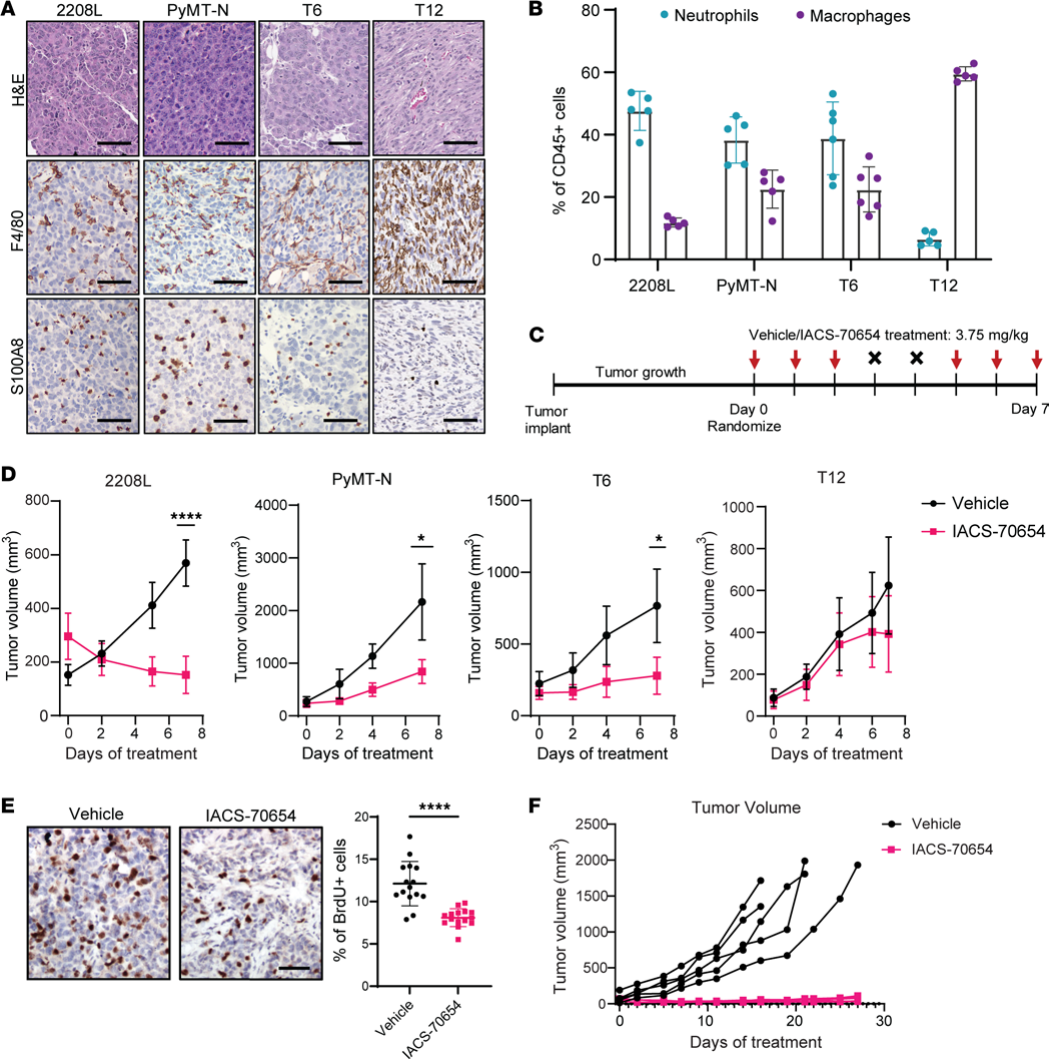
IACS-70654 suppressed the growth of neutrophil-enriched preclinical mouse models of TNBC. (**A**) Representative images of H&E and immunostaining of all TNBC preclinical mouse models used. F4/80 is a macrophage marker, and S100A8 is a neutrophil marker. Scale bars: 50 μm. (**B**) Flow cytometry analysis of TANs and TAMs in all TNBC preclinical mouse models used (T6, *n* = 6; all other models, *n* = 5). (**C**) The treatment scheme of IACS-70654 in a 7-day experiment. When the average tumor volume reached 80–250 mm^3^, the mice were randomized to initiate treatment. Vehicle or IACS-70654 at 3.75 mg/kg was administered orally on a 3-on/2-off schedule. On day 7, mice were euthanized 2 hours after receiving the last treatment. (**D**) Tumor growth curves of all TNBC models treated with IACS-70654 for 7 days (2208L, *n* = 6; all other models, *n* = 5). Two-way ANOVA and Šidák’s multiple-comparison test were used. (**E**) Left: Representative images of immunostaining of BrdU in 2208L tumor sections treated with vehicle or IACS-70654 for 7 days. Scale bar: 50 μm. Right: Quantification of BrdU staining (*n* = 15). A 2-tailed, unpaired Student’s *t* test was used. For **B**, **D**, and **E**, data are presented as mean ± SD. *P < 0.05; ****P < 0.0001. The experiments were conducted 3 times for 2208L and twice for other models. Data from 1 representative experiment are shown. (**F**) Tumor growth curves (from 1 representative experiment) of 2208L tumors treated with IACS-70654 for 27 days. The experiment was conducted twice.

To examine the effects of CBP/P300 BRD inhibition on TNBC in vivo, we selected 4 immunocompetent mouse models derived from the BALB/c or C57BL/6J background. T6, T12, and 2208L tumors are syngeneic *Trp53*-null models, which were shown to recapitulate the aggressiveness, heterogeneity, and resistance to standard-of-care therapies in human TNBC (24–26). PyMT-N is a luminal-like subtype derived from MMTV-PyMT tumors, exhibiting stable myeloid cell infiltration and resistance to immune checkpoint blockade (ICB) (7). These models displayed distinct TAN and TAM frequencies. TANs are defined as CD45^+^CD11b^+^Ly6G^+^Ly6C^lo/med^ in flow cytometry and S100A8^+^ in immunostaining. TAMs are defined as CD45^+^CD11b^+^Ly6G^–^Ly6C^–^F4/80^+^ in flow cytometry and F4/80^+^ in immunostaining. PyMT-N, 2208L, and T6 can be categorized as neutrophil-enriched models, and T12 can be categorized as a macrophage-enriched model (7, 27) (Figure 1, A and B). Tumor pieces (*Trp53-*null models) or freshly dissociated tumor cells (PyMT-N) were implanted into the mammary fat pad of BALB/c or C57BL/6J mice. When the tumors reached more than 80 mm^3^ in volume on average, the mice were randomized, and treatment was initiated (Figure 1C). IACS-70654 is a potent and highly selective inhibitor of CBP/P300 BRD (Supplemental Tables 1 and 2 and Supplemental Figure 1A; supplemental material available online with this article; https://doi.org/10.1172/jci.insight.182621DS1). IACS-70654 was orally administered at the dosage of 3.75 mg/kg on a 3-on/2-off regimen for all animal studies, and therefore a total of 6 doses were given in a 7-day experiment (Figure 1C). IACS-70654 treatment was well tolerated and did not lead to significant body weight loss (Supplemental Figure 1B). Surprisingly, IACS-70654 as a single agent resulted in the regression of 2208L tumors, which has the highest TAN frequency (Figure 1, B and D, and Supplemental Figure 1C). Tumor regression is rarely observed with a single-agent treatment in *Trp53-*null models because they are highly aggressive and resistant to therapies. IACS-70654 reduced the growth of PyMT-N and T6 tumors, which are neutrophil enriched but infiltrated with more TAMs than 2208L tumors (Figure 1, B and D, and Supplemental Figure 1C). The macrophage-enriched T12 model was resistant (Figure 1, B and D, and Supplemental Figure 1C). We also used BrdU to determine the effect of IACS-70654 on tumor cell proliferation. 2208L tumors treated with IACS-70654 showed a reduction in BrdU incorporation and thus were less proliferative (Figure 1E). To test whether tumor inhibition can persist in long-term treatment, we conducted a 27-day treatment study of IACS-70654 with 2208L tumors. IACS-70654 durably inhibited the growth of 2208L tumors, with no signs of resistance (Figure 1F). Histone H3 lysine 27 acetylation (H3K27ac) is the target biomarker for IACS-70654, and reduced H3K27ac was observed after in vitro and in vivo treatments with IACS-70654 (Supplemental Figure 1, D–F). IACS-70654 did not affect the expression of CBP, P300, or the RNA expression of other major epigenetic enzymes (Supplemental Figure 1, E–G). Based on those findings, we hypothesized that the infiltrated myeloid cells might explain the difference in response. Therefore, we next examined the changes in infiltrated myeloid cells after IACS-70654 treatment.

### IACS-70654 reduced TANs and immunosuppression in the TIME.

**Figure 2:**
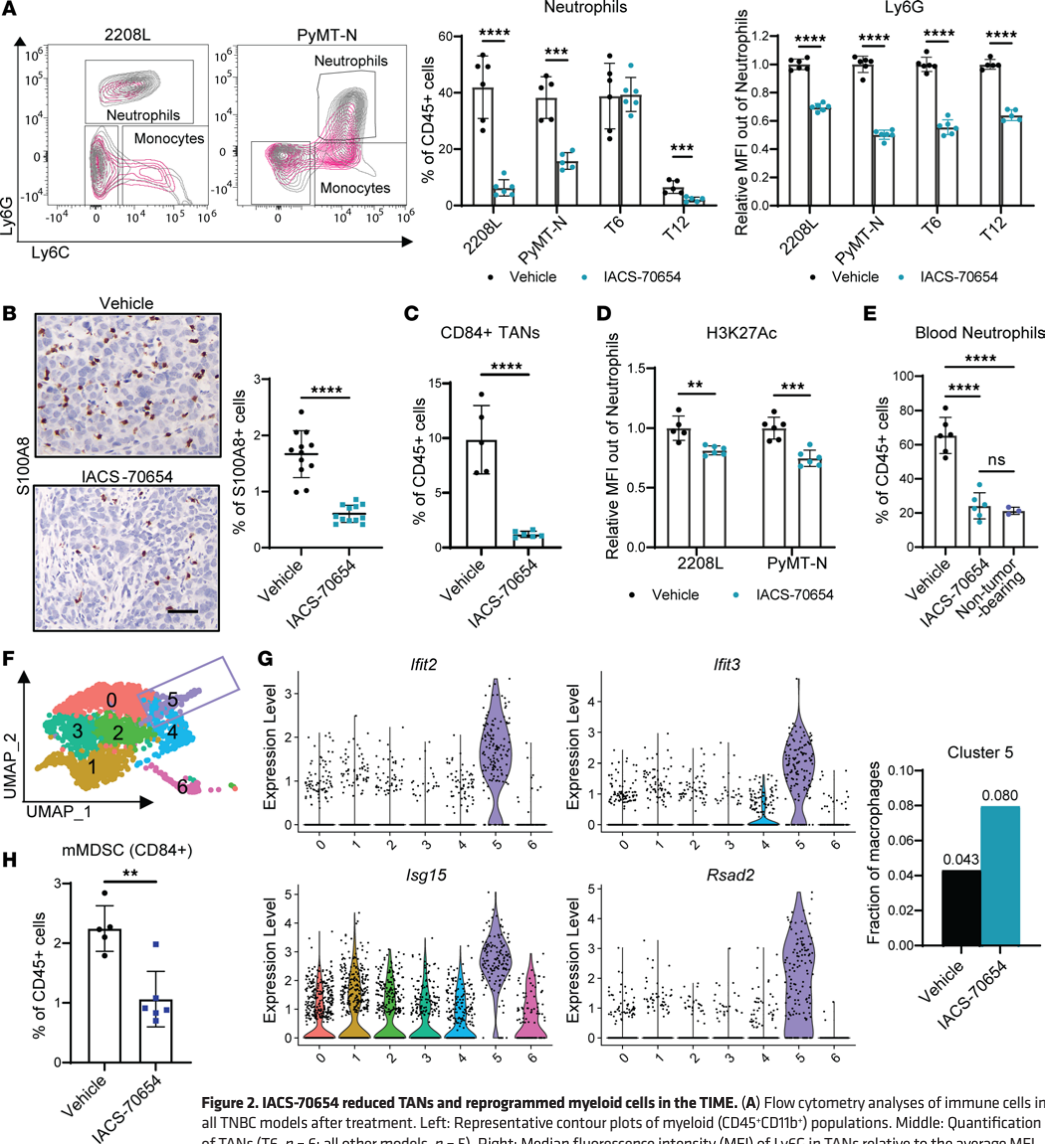
IACS-70654 reduced TANs and reprogrammed myeloid cells in the TIME. (**A**) Flow cytometry analyses of immune cells in all TNBC models after treatment. Left: Representative contour plots of myeloid (CD45^+^CD11b^+^) populations. Middle: Quantification of TANs (T6, *n* = 6; all other models, *n* = 5). Right: Median fluorescence intensity (MFI) of Ly6G in TANs relative to the average MFI of the vehicle-treated group (T6 and PyMT-N, *n* = 6; 2208L and T12, *n* = 5). (**B**) Left: Representative images of S100A8 immunostaining on 2208L tumor sections. Scale bar: 50 μm. Right: Quantification of S100A8 staining (*n* = 12). For **A** and **B**, experiments were conducted 3 times for 2208L and twice for other models. Data from 1 representative experiment are shown. (**C**) Flow cytometry analysis of CD84^+^ TANs in 2208L tumors after treatment (*n* = 5). (**D**) MFI of H3K27ac in TANs relative to the average MFI of the vehicle-treated group in 2208L and PyMT-N tumors after treatment (2208L, *n* = 5; PyMT-N, *n* = 6). (**E**) Flow cytometry analyses of blood neutrophils in 2208L tumor–bearing mice after treatment and non–tumor-bearing BALB/c mice. Ordinary 1-way ANOVA and Tukey’s multiple-comparison test were used (2208L tumor–bearing mice, *n* = 5; non–tumor-bearing mice, *n* = 3). (**F**) UMAP plot of TAM population (monocytes included). (**G**) Left: Violin plot showing the expression of representative IFN-associated genes across TAM clusters. Right: Fraction of Cluster 5 in TAMs. The fraction values are labeled. (**H**) Flow cytometry analysis of tumor-associated mMDSCs (Ly6G^–^Ly6C^+^CD84^+^) in 2208L tumors after treatment (vehicle, *n* = 5; IACS-70654, *n* = 6). For **A**–**D** and **H**, a 2-tailed, unpaired Student’s *t* test was used. For **A**–**E** and **H**, data are presented as mean ± SD. **P < 0.01; ***P < 0.001; ****P < 0.0001. For **A**–**H**, tumors were treated with vehicle or IACS-70654 for 7 days.

To investigate the effects of IACS-70654 on tumor-infiltrated myeloid cells, we analyzed the infiltrated immune cells of the selected models from the 7-day treatment study. IACS-70654 reduced Ly6G expression in TANs from all 4 models and significantly reduced the percentage of TANs in 2208L, PyMT-N, and T12 tumors (Figure 2A and Supplemental Figure 2A). The reduction in TANs was verified with reduced immunostaining of S100A8 in 2208L tumors and was observed within 72 hours after starting treatment (Figure 2B and Supplemental Figure 2B). TANs of neutrophil-enriched models were demonstrated to be immunosuppressive (7). To verify, we applied a published MDSC gene signature to TANs from single-cell RNA sequencing (scRNA-seq) analyses of 2208L tumors treated with IACS-70654 for 7 days (Supplemental Table 3) (5). TANs from 2208L tumors showed higher expression of the MDSC gene signature than the tumor-associated monocytes, and the expression decreased with IACS-70654 treatment (Supplemental Figure 2C). Furthermore, CD84 is an emerging marker used to distinguish PMN-MDSCs from neutrophils, and IACS-70654 substantially reduced CD84^+^ TANs (PMN-MDSCs) in 2208L tumors (Figure 2C) (4, 5). In contrast, TANs of macrophage-enriched tumors were shown to resemble normal neutrophils (7). Therefore, reduced TANs should not reduce immunosuppression in T12 tumors, and thus this may partially explain why T12 did not respond to IACS-70654. Higher TAM frequency in PyMT-N and T6 might explain why regression was only observed in 2208L tumors. TANs in 2208L and PyMT-N tumors treated with IACS-70654 were also found to have reduced expression of H3K27ac, without changes in RNA expression of genes encoding CBP and P300 (Figure 2D and Supplemental Figure 2D). This result suggested that TANs can be directly reprogrammed by IACS-70654. Moreover, high TAN frequency is accompanied by an accumulation of peripheral blood neutrophils (7, 8). IACS-70654 reduced blood neutrophils in 2208L tumor–bearing mice to a similar level as non–tumor-bearing mice (Figure 2E). We also treated non–tumor-bearing mice with IACS-70654 and observed no significant change in blood neutrophils (Supplemental Figure 2E).

Besides neutrophils, IACS-70654 also affected other infiltrated myeloid cells. Even in neutrophil-enriched tumors, TAMs remain the second most abundant immune cell, indicating their importance (7) (Figure 1B). TAMs can have various functions, leading to an antitumor or protumor phenotype (28). To determine transcriptional changes in TAMs, we analyzed TAMs from the scRNA-seq of 2208L tumors treated with vehicle or IACS-70654 for 7 days. Six TAM clusters with distinct expression profiles were identified (Figure 2F, Supplemental Figure 2, F and G, and Supplemental Tables 6–12). Cluster 5 of TAMs highly expressed IFN-response genes and pathways, which are associated with the antitumor phenotype (29) (Figure 2G, Supplemental Figure 2H, Supplemental Table 4, and Supplemental Table 5). IACS-70654 treatment led to an almost 2-fold increase in the fraction of cluster 5 (Figure 2G). Moreover, monocytes are defined as CD45^+^CD11b^+^Ly6G^–^Ly6C^+^, and those that express CD84 can be considered monocytic myeloid-derived suppressor cells (mMDSCs) (4, 5). IACS-70654 reduced mMDSC infiltration in 2208L and PyMT-N tumors (Figure 2H). Arginase 1 (Arg) is also a marker of immunosuppression, and IACS-70654 reduced Arg^+^ monocytes/mMDSCs in 2208L tumors (Supplemental Figure 2I). These results suggested that IACS-70654 can reduce immunosuppression and might be an effective therapy for neutrophil-enriched TNBC, which has been correlated with poor patient outcomes and therapy resistance (3, 7). Because IACS-70654 reduced neutrophils in tumors and blood, we further hypothesized that IACS-70654 might inhibit the abnormal neutrophil generation in BM promoted by tumor outgrowth (8–10).

### IACS-70654 reprogrammed and reduced the proliferation of neutrophils in BM.

**Figure 3:**
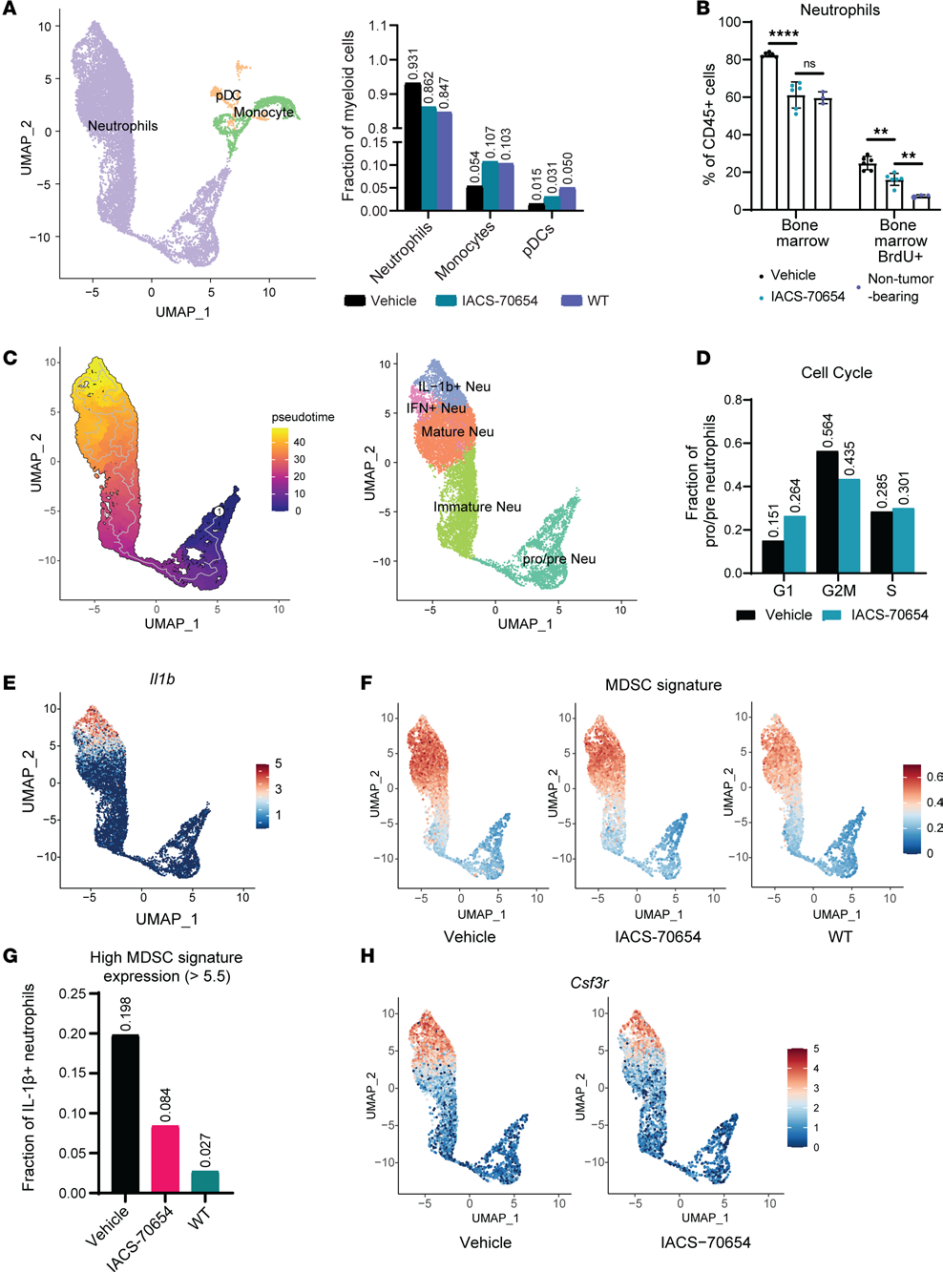
IACS-70654 reprogrammed BM neutrophils. (**A**) Left: UMAP plot of BM myeloid cells with annotations. Right: Fractions of BM myeloid cells in treated 2208L tumor–bearing and untreated non–tumor-bearing WT BALB/c mice from scRNA-seq analyses. (**B**) Representative flow cytometry analyses of neutrophils in the BM of treated 2208L tumor–bearing mice (7-day treatment with vehicle or IACS-70654) and untreated non–tumor-bearing WT BALB/c mice (2208L tumor–bearing mice, *n* = 5; non–tumor-bearing mice, *n* = 3). Ordinary 1-way ANOVA and Tukey’s multiple-comparison test were used. **P < 0.01; ****P < 0.0001; NS, *P* > 0.05. Data are presented as mean ± SD. The experiment was conducted twice. (**C**) Left: Pseudotime analysis of integrated BM neutrophils. The root is circled. Right: UMAP plot of BM neutrophils with annotations. (**D**) Fractions of pre-neutrophils and pro-neutrophils in G_1_, G_2_/M, or S cell cycle stage in the BM of 2208L tumor–bearing mice after treatment from scRNA-seq analyses. (**E**) Expression distribution of *Il1b* in integrated BM neutrophils. (**F**) Expression distribution of the MDSC gene signature in BM neutrophils of treated 2208L tumor–bearing mice and untreated non–tumor-bearing mice (WT). (**G**) Fraction of IL-1β^+^ neutrophils that have a high expression level (>0.55) of the MDSC gene signature in treated 2208L tumor–bearing mice and untreated non–tumor-bearing mice (WT). (**H**) Expression distribution of *Csf3r* in BM neutrophils of treated 2208L tumor–bearing mice and untreated non–tumor-bearing mice (WT). For **A** and **C**–**H**, 2208L tumor–bearing mice were treated with vehicle or IACS-70654 for 6 days.

To characterize the changes in BM after IACS-70654 treatment, we first performed scRNA-seq analyses on all CD45^+^ cells collected from BM of non–tumor-bearing WT and 2208L tumor–bearing mice. The 2208L tumor–bearing mice were treated with vehicle or IACS-70654 for 6 days (5 total treatments). As expected, the fraction of neutrophils was increased in 2208L tumors, with a concomitant decrease in the fraction of monocytes and dendritic cell progenitors (pDCs) (Figure 3A). IACS-70654 treatment reduced BM production of neutrophils and restored that of monocytes and pDCs (Figure 3A). This observation was confirmed by flow cytometry analyses coupled with measuring BrdU incorporation (Figure 3B and Supplemental Figure 3A). Reduced H3K27ac was observed in the CD11b^+^Ly6G^lo/med^F4/80^–^ neutrophil precursors (Supplemental Figure 3B). Neutrophils were then isolated and clustered to identify subpopulations using pseudotime analysis and previously identified markers such as *Itgam* and *Cxcr2* expression (26, 30, 31) (Figure 3C and Supplemental Figure 3C). Pro-neutrophils (proNeu) and Pre-neutrophils (preNeu) are the proliferative precursors (31). We examined the cell cycle score of the proliferative proNeu/preNeu and found that IACS-70654 decreased the fraction of cells exhibiting a G_2_/M signature (Figure 3D). Previous studies showed BM neutrophils in tumor-bearing mice are immunosuppressive, especially in those with advanced tumors (7, 32). Among the mature neutrophils, we identified a distinct subpopulation with high expression of IL-1β, an MDSC marker, and this subpopulation was upregulated in 2208L tumor–bearing mice compared with the non–tumor-bearing mice (4) (Figure 3E and Supplemental Figure 3D). BM mature neutrophils in 2208L tumor–bearing mice also expressed a higher MDSC signature than those in non–tumor-bearing mice, and the expression decreased with IACS-70654 treatment (Figure 3, F and G). We further verified that IACS-70654 reduced CD84^+^ BM neutrophils in 2208L tumor–bearing mice using flow cytometry (Supplemental Figure 3E). In addition to the immunosuppressive neutrophils, a previous study identified IFN-stimulated neutrophils (IFN+ Neu) in blood. Increased IFN+ Neu was correlated with a better response of mouse tumor models and cancer patients to immunotherapy (33). We applied the published gene signature and identified IFN+ Neu in BM neutrophils (Supplemental Figure 3F and Supplemental Table 13). IACS-70654 increased the fraction of IFN+ Neu (Supplemental Figure 3D). Moreover, IACS-70654 reduced the expression of *Csf3r*, which encodes the receptor for granulocyte colony–stimulating factor (G-CSF), in mature neutrophils (Figure 3H, Supplemental Table 16, and Supplemental Table 18). G-CSF level was upregulated by the 2208L tumor (Supplemental Figure 3G). IACS-70654 might reduce G-CSF signaling, a critical driver for neutrophil production, migration, and immunosuppression (8, 34, 35). IACS-70654 also decreased the RNA expression of genes encoding CCR1 and Ly6G, both of which are critical for neutrophil migration (36, 37) (Supplemental Figure 3, H and I, Supplemental Table 14, Supplemental Table 16, and Supplemental Table 18). In addition, we discovered that BM neutrophils in 2208L tumor–bearing mice downregulated the expression of *Thbs1*, which encodes thrombospondin-1 (TSP-1), and IACS-70654 restored the expression (Supplemental Figure 3J). The expression of cystatins (e.g., *Stfa2*, *Stfa3*, *Cstdc5*, *Cstdc6*) and hydrolase/peptidase activity inhibition pathways were also significantly upregulated in BM neutrophils after IACS-70654 treatment (Supplemental Figure 3K and Supplemental Tables 14–19). TSP-1 and cystatins are both inhibitors of neutrophil serine protease that were reported to promote neutrophil release into the blood (38, 39). They can also contribute to the release of C-X-C motif chemokine ligand 2, which promotes neutrophil recruitment, and activates IL-1β (40, 41). These results revealed that IACS-70654 might reduce the proliferation, migration, and protumor activity of BM neutrophils. However, hematopoietic stem and progenitor cells (HSPCs) may also be affected by tumor-derived factors (8, 9). Accordingly, we next investigated the changes in HSPCs after IACS-70654 treatment.

### IACS-70654 induced transcriptional changes in HSPCs to reduce abnormal myelopoiesis.

**Figure 4:**
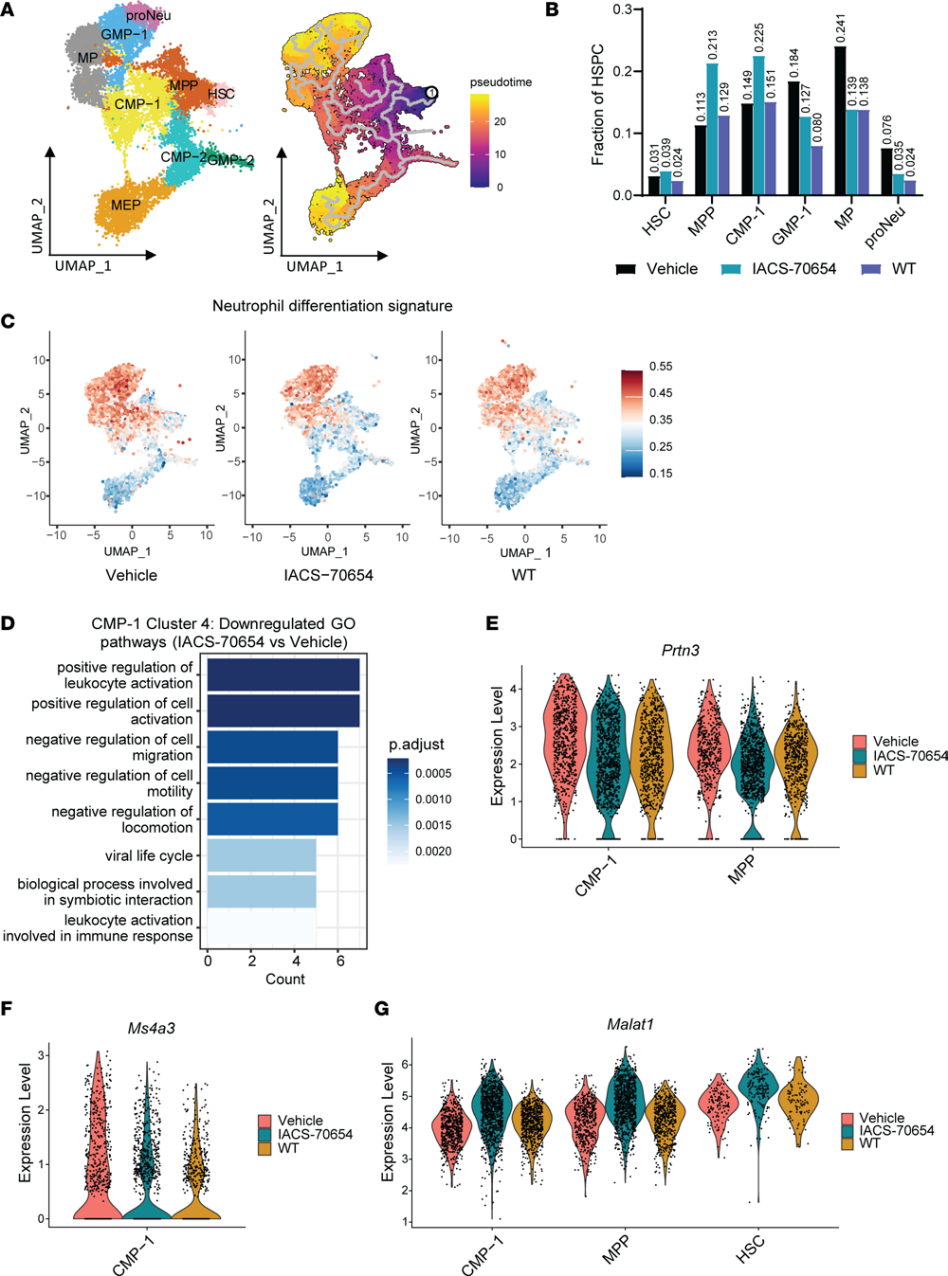
IACS-70654 reprogrammed abnormal myelopoiesis induced by the neutrophil-enriched tumor. (**A**) UMAP plot with annotations (left) and pseudotime analysis (right) of integrated HSPC clusters. (**B**) Fraction of myeloid progenitor cells in HSPCs of treated 2208L tumor–bearing mice and untreated non–tumor-bearing WT mice. (**C**) Expression distribution of neutrophil differentiation signature from scRNA-seq analyses of HSPCs in treated 2208L tumor–bearing mice and untreated non–tumor-bearing WT mice. (**D**) GO pathway enrichment analysis of the significantly downregulated genes in cluster 4 (annotated as CMP-1) of HSPCs of 2208L tumor–bearing mice treated with IACS-70654 versus vehicle. Biological Process (BP) gene sets from the GO database were used. The top 8 pathways are listed. (**E**) Violin plots showing the RNA expression of *Prtn3* in CMP-1 and MPPs of treated 2208L tumor–bearing mice and untreated non–tumor-bearing WT mice. (**F**) Violin plot showing the RNA expression of *Ms4a3* in CMP-1 of treated 2208L tumor–bearing mice and untreated non–tumor-bearing WT mice. (**G**) Violin plots showing the RNA expression of *Malat1* in CMP-1, MPPs, and HSCs from treated 2208L tumor–bearing mice and untreated non–tumor-bearing WT mice. For **A**–**G**, 2208L tumor–bearing mice were treated with vehicle or IACS-70654 for 6 days.

To determine whether IACS-70654 can reprogram the abnormal myelopoiesis induced by the neutrophil-enriched tumor, we performed scRNA-seq on HSPCs in BM of 2208L tumor–bearing mice treated with vehicle or IACS-70654 and non–tumor-bearing mice. The dataset was filtered to contain only HSPCs involved in myelopoiesis and annotated using classic HSPC markers, as described previously (9, 42). Two distinct populations of granulocyte-monocyte progenitors (GMPs) and common myeloid progenitors (CMPs) were found (Figure 4A and Supplemental Figure 4A). Based on clustering and trajectory analyses, GMP-1 and CMP-1 were determined to be involved in myelopoiesis (Figure 4A). 2208L tumors upregulated the fraction of GMP-1, monocyte progenitors (MPs), and proNeu, whereas IACS-70654 treatment reduced those progenitors, indicating inhibition of myelopoiesis (Figure 4B). We also examined the expression of a published gene signature that was shown to predict the differentiation of HSPCs toward neutrophils (43) (Supplemental Table 20). HSPCs, especially CMP-1 and multipotent progenitors (MPPs), showed higher expression of the gene signature in 2208L tumor–bearing mice than those in non–tumor-bearing WT mice, and the expression decreased with IACS-70654 treatment (Figure 4C, Supplemental Figure 4B, and Supplemental Tables 23–26). In cluster 4, a CMP-1 cluster, IACS-70654 led to the downregulation of myeloid differentiation and activation pathways (Figure 4D, Supplemental Table 21, and Supplemental Table 22). In addition, IACS-70654 decreased the expression of *Prtn3* and *Ms4a3* in CMP-1 (Figure 4, E and F, and Supplemental Table 23). The expression of these genes has been associated with the differentiation and proliferation of myeloid progenitor cells and found to be increased in the granulocytes from the peripheral blood of TNBC patients (9, 44, 45). These changes imply that IACS-70654 might reduce the differentiation of HSPCs into neutrophils. Moreover, in CMP-1, MPPs, and hematopoietic stem cells (HSCs), IACS-70654 induced the expression of *Malat1*, a long noncoding RNA shown to inhibit differentiation of early HSPCs (46) (Figure 4G, Supplemental Table 23, Supplemental Table 25, and Supplemental Table 26). In HSCs and MPPs, IACS-70654 also increased the expression of *Txnip*, which can keep HSCs in an undifferentiated state and reduce their mobility (47) (Supplemental Figure 4C, Supplemental Table 25, and Supplemental Table 26). Furthermore, in the peripheral blood of 2208L tumor–bearing mice, IACS-70654 reduced the level of IL-3, a cytokine known to induce HSC and myeloid differentiation (48, 49) (Supplemental Figure 4D). These observations suggest that IACS-70654 treatment might retain the early HSPCs in an undifferentiated state, explaining the accumulation of those progenitors (Figure 4B). Taken together, these results suggested that IACS-70654 reduced the differentiation of early HPSCs to reduce the overproduction of GMP-1 and thus neutrophils. Besides neutrophils, IACS-70654 may also elicit effects on 2208L tumor cells. Thus, we next investigated the effects of IACS-70654 in tumor cells.

### IACS-70654 induced both an IFN response and antigen presentation in tumor cells.

**Figure 5:**
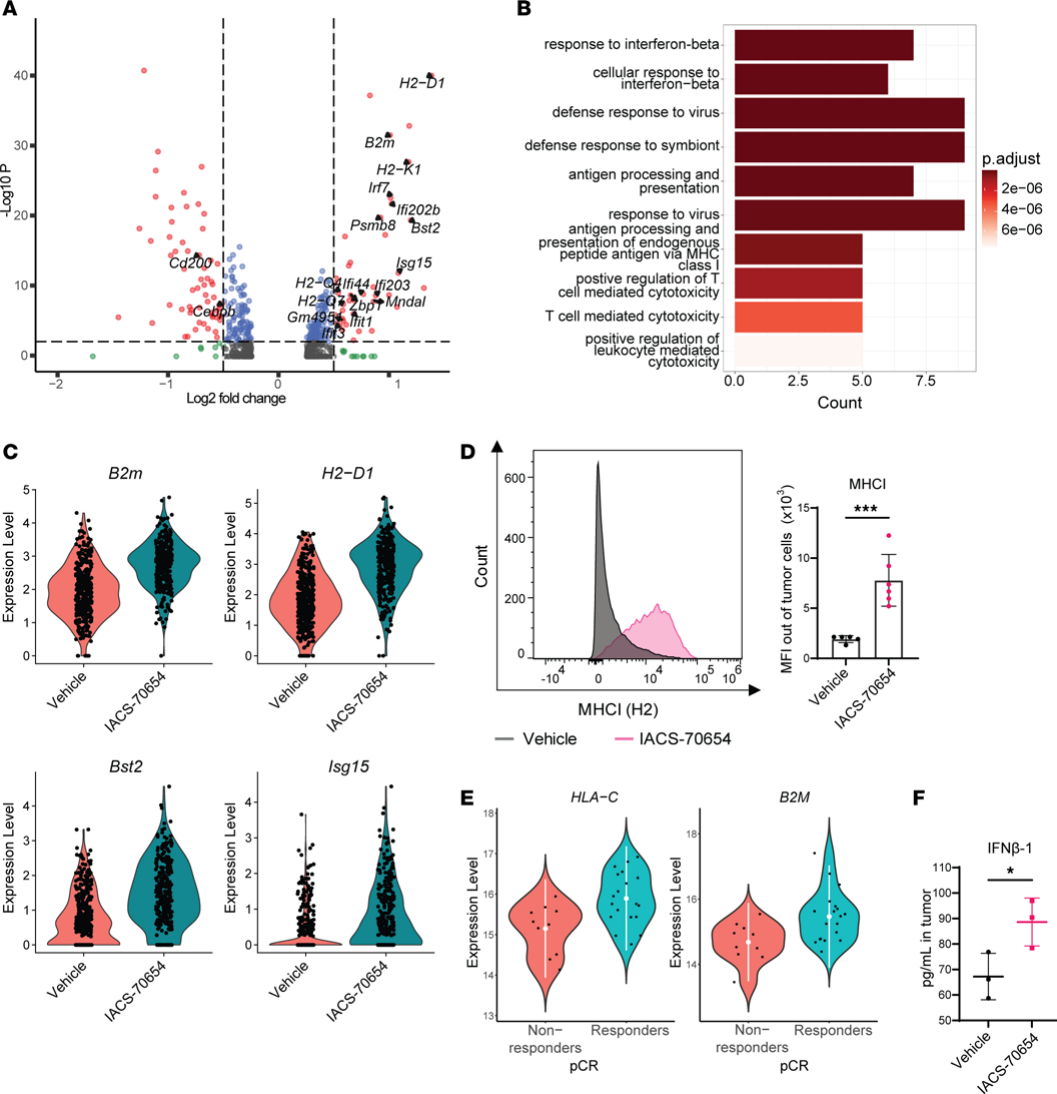
IACS-70654 induced an IFN-associated response and MHCI expression in 2208L tumor cells. (**A**) Volcano plot showing –log_10_(*P* value) versus log_2_(fold change) in RNA expression in 2208L tumor cells treated in vivo with IACS-70654 versus those treated with vehicle. Genes demonstrating significant changes are represented by red dots. Genes associated with IFN response, antigen representation, and immunosuppression are labeled. (**B**) GO pathway enrichment analysis of the significantly upregulated genes in 2208L tumor cells treated in vivo with IACS-70654 versus those treated with vehicle. BP gene sets were used. The top 10 pathways are listed. (**C**) Violin plots showing RNA expression of representative MHCI components (*B2m* and *H2-D1*) and IFN response genes (*Bst2* and *Isg15*) in 2208L tumor cells treated in vivo. (**D**) Flow cytometry analysis of MHCI expression in 2208L tumor cells treated in vivo. Left: Representative histograms of MHCI expression in 2208L tumor cells (CD45^–^TER119^–^CD31^–^EpCAM^+^). Right: MFI of MHCI in 2208L tumor cells (vehicle arm, *n* = 5; IACS-70654 arm, *n* = 6). (**E**) Violin plots showing RNA expression (mean ± 2 SD) of representative MHCI components *B2M* (meta *P* = –0.0373) and *HLA-C* (meta *P* = –0.006612) in human TNBC patients who did (responders) or did not (non-responders) achieve pathological complete response (pCR) after treatment of paclitaxel and pembrolizumab (anti–PD-1). Data were retrieved from published studies (50, 51). (**F**) Quantification of IFN-β level in 2208L tumor homogenate by cytokine/chemokine array (*n* = 3). For **D** and **F**, a 2-tailed, unpaired Student’s *t* test was used. *P < 0.05; ***P < 0.001. Data are presented as mean ± SD. For **A**–**D** and **F**, 2208L tumors were treated with vehicle or IACS-70654 for 7 days.

To investigate how IACS-70654 impacts tumor cells, we first analyzed the tumor cell cluster from scRNA-seq of 2208L tumors treated with vehicle or IACS-70654 for 7 days (Supplemental Figure 5, A and B). IACS-70654 induced the expression of MHCI components (e.g., *H2-D1*, *H2-K1*, *H2-Q4*) (Figure 5, A–C, Supplemental Table 27, and Supplemental Table 28). Using flow cytometry, we confirmed that IACS-70654 treatment induced the protein expression of MHCI on the surface of 2208L tumor cells in vivo (Figure 5D). Higher expression of MHCI component genes such as *B2M* and *HLA-C* and the antigen processing and presentation pathway is associated with pathological complete response (pCR) after paclitaxel and pembrolizumab (ICB) treatment in TNBC patients (Figure 5E, Supplemental Figure 5D, and Supplemental Table 29) (50, 51). Besides MHCI, IACS-70654 also induced the expression of genes associated with IFN-β– and virus-response pathways (Figure 5, A–C, Supplemental Table 27, and Supplemental Table 28). Using a cytokine/chemokine array, we detected an elevated level of IFN-β in 2208L tumors treated with IACS-70654 compared with those treated with vehicle (Figure 5E). IACS-70654 also downregulated genes involved in the regulation of inflammation and inhibition of cytokine production, such as *Cd200* and *Cebpb* that have been shown to induce immunosuppression in the TIME (52, 53) (Figure 5A, Supplemental Figure 5C, and Supplemental Table 27). These results demonstrated that IACS-70654 induced both MHCI expression and an IFN response in 2208L tumor cells, suggesting induced antigen presentation and stimulation of the immune response. Therefore, we next investigated the effects of IACS-70654 on tumor-infiltrated lymphocytes and the response of neutrophil-enriched tumors to ICB.

### IACS-70654 activated cytotoxic T cells and improved response to ICB.

**Figure 6:**
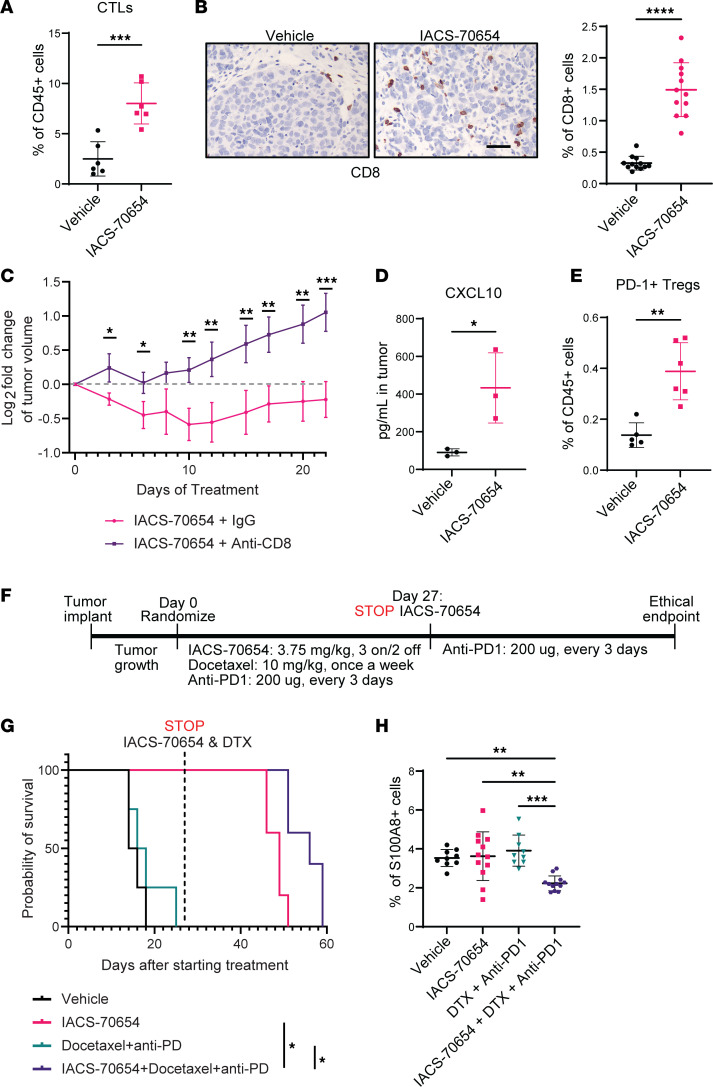
IACS-70654 induced a CTL-dependent response and improved response to ICB. (**A**) Flow cytometry analysis of CTLs in 2208L tumors treated for 7 days (*n* = 6). (**B**) Left: Representative images of CD8 immunostaining on sections of 2208L tumors treated for 7 days. Scale bar: 50 μm. Right: Quantification of CD8 staining (*n* = 12). For **A** and **B**, experiments were conducted 3 times. Data from 1 representative experiment are shown. (**C**) Log_2_(fold change) of 2208L tumor volume (mean ± SD). Anti-CD8 was administered 24 hours before starting IACS-70654 treatment. Two-way ANOVA and Šidák’s multiple-comparison test were used (*n* = 5). (**D**) Quantification of CXCL10 level in 2208L tumor homogenate by cytokine/chemokine array (*n* = 3). Tumors were treated for 7 days. (**E**) Flow cytometry analysis of Tregs in 2208L tumors treated for 18 days (*n* = 5). For **A**, **B**, **D**, and **E**, a 2-tailed, unpaired Student’s *t* test was used. (**F**) Treatment schemes of IACS-70654 in combination with anti–PD-1 and DTX. DTX was administered at half of the clinically equivalent dose. After 27 days of treatment, only anti–PD-1 treatment was administered until all tumors reached the ethical endpoint (≥1500mm^3^). (**G**) Kaplan-Meier survival curves of 2208L tumor–bearing mice (vehicle and DTX + anti–PD-1 arms, *n* = 4; IACS-70654 and combination arms, *n* = 5). Log-rank test with Bonferroni’s correction was used. *P < 0.05. The experiment was conducted twice. Data from 1 representative experiment are shown. (**H**) Quantification of S100A8 immunostaining. Ordinary 1-way ANOVA and Šidák’s multiple-comparison test were used (vehicle and DTX + anti–PD-1 arms, *n* = 9; IACS-70654 and combination arms, *n* = 12). *P < 0.05; **P < 0.01; ***P < 0.001; ****P < 0.0001. For **A**–**E** and **H**, data are presented as mean ± SD.

Neutrophil-enriched TNBC models such as 2208L usually have low lymphocyte infiltration and complete resistance to ICB (7). After a 7-day treatment with IACS-70654, 2208L tumors showed a significantly higher level of cytotoxic T cell (CTL, defined as CD3^+^CD8^+^) infiltration (Figure 6A). This finding was confirmed using immunostaining, and CTLs were observed in the tumor center instead of the stroma after IACS-70654 treatment (Figure 6B). To determine whether CTLs play a critical role in tumor growth inhibition by IACS-70654, 2208L tumor–bearing mice were treated with an anti-CD8 antibody 24 hours before starting IACS-70654 treatment and then throughout the experiment. Successful depletion of tumor-infiltrated CTLs was confirmed by flow cytometry (Supplemental Figure 6A). CTL depletion attenuated the effects of IACS-70654 and suggested that CTLs were important in both the immediate tumor regression and the durable inhibition of tumor growth (Figure 6C). Moreover, in 2208L tumors, IACS-70654 induced higher levels of CXCL10, a chemokine contributing to CTL recruitment and associated with better efficacy of ICB (Figure 6D) (54). IACS-70654 did not increase programmed cell death protein 1^+^ (PD-1^+^) CTLs, but increased PD-1^+^ regulatory T cells (Tregs, defined as CD4^+^FoxP3^+^), implying a potential benefit of combining IACS-70654 with anti–PD-1 (Figure 6E and Supplemental Figure 6B). Thus, we tested the efficacy of IACS-70654 in combination with docetaxel (DTX) and anti–PD-1, which partially mimics the current standard-of-care therapy for TNBC (Figure 6F). DTX is known to cause adverse effects in breast cancer patients, and lowering the dose is commonly used to mitigate adverse effects (55, 56). Therefore, in this study, DTX was administered at 10 mg/kg, which is half of the clinically relevant dose. The combination treatment was well tolerated and showed no signs of toxicity (Supplemental Figure 6C). DTX in combination with anti–PD-1 failed to inhibit tumor growth, and all tumors treated with vehicle or DTX in combination with anti–PD-1 reached the ethical endpoint within 25 days (Figure 6G and Supplemental Figure 6D). IACS-70654 in combination with DTX and anti–PD-1, similarly to IACS-70654 alone, durably inhibited the tumor growth (Supplemental Figure 6D). To determine whether the combination treatment has durable long-term effects on the response of 2208L tumors to anti–PD-1, we stopped IACS-70654 and DTX treatment on day 27, continuing only anti–PD-1 treatment (Figure 6G). Compared with IACS-70654 alone, IACS-70654 in combination with DTX and anti–PD-1 significantly delayed the regrowth of 2208L tumors (Figure 6G). Furthermore, immunostaining of the tumors at the endpoint revealed that TANs again accumulated in the tumors treated with IACS-70654, indicating immunosuppression (Figure 6H). However, tumors treated with IACS-70654 in combination with DTX and anti–PD-1 were infiltrated with significantly fewer TANs, which might explain the delayed recurrence (Figure 6H). In summary, IACS-70654 inhibited the growth of 2208L tumors in a CTL-dependent manner and can potentially improve the response of neutrophil-enriched TNBC to standard-of-care therapies. Although we demonstrated the efficacy of IACS-70654 treatment in primary tumors of neutrophil-enriched TNBC models, the clinically unmet need is to treat metastasis. Accordingly, we next investigated the effects of IACS-70654 in the metastatic setting.

### IACS-70654 inhibited the growth of established lung metastases.

**Figure 7:**
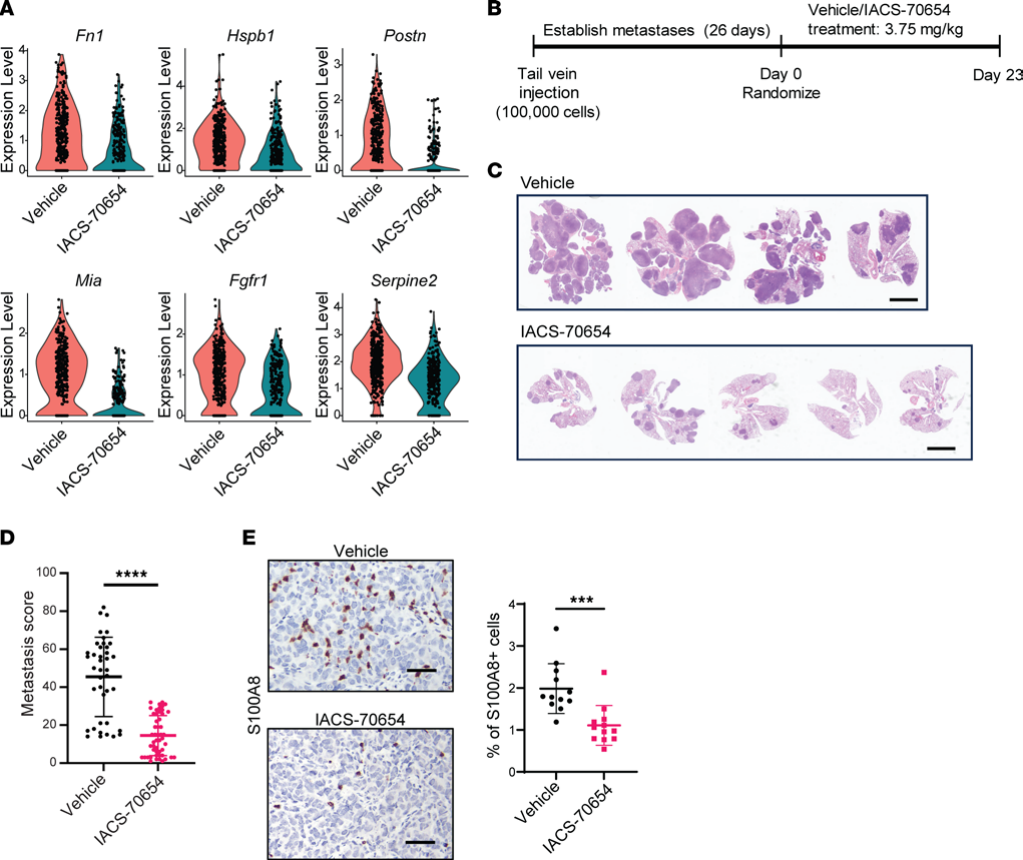
IACS-70654 reduced the growth of established 2208L lung metastases. (**A**) Violin plots showing RNA expression of representative genes that might be involved in migration and metastasis in 2208L tumor cells treated in vivo. Adjusted *P* < 0.01 for all genes. (**B**) Experimental design for studying the effects of IACS-70654 on established lung metastases. Dissociated 2208L tumor cells (100,000) were injected into the tail vein of each mouse. The mice were treated for 23 days. One mouse from the vehicle group had to be euthanized on day 22 due to poor body condition. (**C**) Representative images from H&E staining of lungs with 2208L metastases from mice after treatment. Scale bars: 5 mm. (**D**) Quantifications of lung metastases after treatment. Serial sectioning was performed to collect a total of 10 sections for each sample (vehicle, *n* = 40; IACS-70654, *n* = 50). Metastases with sizes of <1 mm, 1–3 mm, 3–5 mm, and >5 mm were assigned scores of 1, 2, 3, and 4, respectively. A 2-tailed, unpaired Student’s *t* test was used. Data are presented as mean ± SD. (**E**) Left: Representative images of S100A8 immunostaining on 2208L lung metastasis sections. Scale bar: 50 μm. Right: Quantification of S100A8 staining (*n* = 12). A 2-tailed, unpaired Student’s *t* test was used. ***P < 0.001; ****P < 0.0001. Data are presented as mean ± SD.

From the scRNA-seq analyses of 2208L tumor cells, we observed that IACS-70654 downregulated the expression of genes associated with migration and negative regulation of cell adhesion pathways (Supplemental Figure 5C and Supplemental Table 28). More importantly, many of the downregulated genes (*Fn1*, *Hspb1*, *Postn*, *Mia*, *Fgfr1*, *Serpine2*) have been associated with tumor metastasis (57–62) (Figure 7A, Supplemental Figure 7A, and Supplemental Table 27). We also observed a reduction in fibroblast growth factor receptor 1 (FGFR1) protein expression in 2208L primary tumors treated with IACS-70654 (Supplemental Figure 7B). Next, we used an experimental metastasis model to test the effects of IACS-70654 on established lung metastases. To generate lung metastases, 100,000 cells dissociated from a 2208L tumor were injected into the tail vein of each WT BALB/c mouse (Figure 7B). Because IACS-70654 was found to affect the immune response, tumor cells were not labeled, since fluorescent reporters may introduce neoantigens (63). We collected the lungs from 2 mice before starting treatment to ensure the successful establishment of lung metastases. The mice were randomized and treated with vehicle or IACS-70654 for 23 days until most vehicle-treated mice reached the ethical endpoint (Figure 7B). We observed that the lung metastatic burden of IACS-70654–treated mice decreased compared with vehicle-treated mice (Figure 7C). The metastatic burden was quantified by counting the metastases using serial sectioning and a size-based scoring system to calculate a metastasis score for each lung section (Figure 7D). The metastasis score of IACS-70654–treated mice was significantly lower than that of vehicle-treated mice (Figure 7D). In the plasma of 2208L lung metastasis–bearing mice, we observed an abnormal upregulation of CCL19, which has been associated with breast cancer metastasis, and the expression level decreased with IACS-70654 treatment (64) (Supplemental Figure 7C). Moreover, neutrophils have been shown to promote metastasis, and 2208L retained its neutrophil-enriched signature in the lung (3) (Figure 7E). IACS-70654 reduced the number of infiltrated neutrophils in 2208L lung metastases (Figure 7E). IACS-70654 also reduced Ly6G expression in the circulating neutrophils of 2208L lung metastasis–bearing mice (Supplemental Figure 7D). Thus, since IACS-70654 inhibited established lung metastasis in these preclinical models, it may potentially provide a therapeutic alternative for the treatment of metastatic neutrophil-enriched TNBC.

## Discussion

This study investigated the effects of CBP/P300 BRD inhibition on 4 syngeneic preclinical models of TNBC using IACS-70654, a selective inhibitor of CBP/P300 BRD. It demonstrated that IACS-70654 inhibited the growth of neutrophil-enriched TNBC models in part by reducing immunosuppressive TANs and activating immune responses.

CBP/P300 BRD inhibition has been reported to reprogram cancer cells to reduce proliferation and treatment resistance in many cancer types, but most studies and clinical trials have focused on blood cancers and prostate cancer (21, 22, 65, 66). For TNBC, CBP/P300 BRD inhibition has been shown to reduce the growth of patient-derived androgen receptor–positive TNBC xenografts and MDA-MB-231 xenografts (23, 67). In contrast, our study reports the effects of CBP/P300 BRD inhibition in syngeneic TNBC models, which are immunocompetent. We confirmed that CBP/P300 BRD inhibition can suppress tumor proliferation in several neutrophil-enriched TNBC models, and our findings suggested that high TAN frequency might predict a better response of TNBC to CBP/P300 BRD inhibitors.

Investigation of the TIME after CBP/P300 BRD inhibition revealed a substantial reduction of TANs in most models tested regardless of TAN frequencies. TANs in neutrophil-enriched models were shown to be immunosuppressive, suggesting that the growth of neutrophil-enriched models might be inhibited after TAN reduction (7). Using a recently published MDSC signature, we confirmed previous findings and showed that IACS-70654 CBP/P300 BRD inhibition reduced PMN-MDSCs (5). The effects of CBP/P300 BRD inhibition on PMN-MDSCs and mMDSCs of TNBC have been studied previously using an MDA-MB-231 xenograft model in immunocompromised hosts, and our observations are in general consistent with what was previously reported (23). However, MDA-MB-231 is a mesenchymal TNBC model highly infiltrated by TAMs, but not TANs, and the macrophage-enriched mesenchymal T12 tumors did not respond significantly to CBP/P300 BRD inhibition (23). This difference may be explained by the nature of a human tumor xenograft model. Since all the stromal cells, including TAMs, are of murine origin, they might lead to weaker support for tumor growth and possibly a less immunosuppressive TIME (68). Moreover, CBP/P300 BRD inhibition certainly does not affect only TANs. In agreement with the previous study, we also observed reduced mMDSCs by CBP/P300 BRD inhibition (23). In addition, we observed an increase in the fraction of a TAM subtype expressing an IFN response signature, which might contribute to immune stimulation and tumor inhibition. Therefore, those changes might be sufficient to reduce tumor growth significantly in a macrophage-enriched xenograft model, but not in a syngeneic model. Overall, our study suggested that CBP/P300 BRD inhibition reduces immunosuppression primarily by reducing TANs, and therefore neutrophil-enriched TNBC may be more sensitive to CBP/P300 BRD inhibition. Furthermore, we have previously observed that several macrophage-enriched TNBC models initially responded to ICB, but rapidly recurred concomitantly with the appearance of immunosuppressive TANs (5). In several of these models, there appears to be a yin yang relationship between immunosuppressive TAMs and TANs. This may provide an escape mechanism for TNBC treated with ICB and suggests that targeting TANs by CBP/P300 BRD inhibition may provide an important therapeutic option.

Previous studies have reported that BM is the primary site responsible for abnormal neutrophil production induced by neutrophil-enriched tumors (4, 8). BM neutrophils from 2208L and PyMT-N tumor–bearing mice were shown previously to suppress T cell activation, and we further confirmed that mature BM neutrophils can have high expression of the MDSC signature (7, 8). CBP/P300 BRD inhibition has been shown to reprogram acute myeloid leukemia and myeloma cells in BM (21). Abnormal proliferation is a shared characteristic between hematological malignancies and neutrophil overproduction caused by neutrophil-enriched TNBC. In this study, we confirmed the overproduction of neutrophils in BM of 2208L tumor–bearing mice and showed that CBP/P300 BRD inhibition reduced the proliferation of neutrophil precursors. CBP/P300 BRD inhibition also reduced the immunosuppressive phenotype in mature neutrophils and increased the number of IFN+ Neu, which are associated with pCR to the standard-of-care therapies in TNBC patients (33). The current study also suggested that CBP/P300 BRD inhibition may disrupt the migration and recruitment of neutrophils. Then, we investigated the early progenitors and determined that the presence of 2208L tumors increased both HSCs and GMP-1, the GMP population shown to differentiate toward neutrophils. HSPCs in tumor-bearing mice also upregulated the gene signature that was shown to predict the differentiation of HSPCs toward neutrophils, suggesting abnormal myelopoiesis (43). These findings agreed with both flow cytometry and scRNA-seq results from our recent study, which demonstrated that the genes associated with the differentiation and proliferation of myeloid progenitors were upregulated, while those associated with the inhibition of myeloid differentiation were downregulated in multiple neutrophil-enriched models, including 2208L and PyMT-N models (9). In the present study, we found that CBP/P300 BRD inhibition reduced GMP-1 and reversed the changes in gene expression induced by the neutrophil-enriched tumor (43). The results also suggested that CBP/P300 BRD inhibition downregulated the differentiation of early progenitors, including CMPs, MPPs, and possibly HSCs, trapping the progenitor cells in an undifferentiated state and leading to reduced GMP-1. Taken together, these results indicate that CBP/P300 BRD inhibition reprogrammed the differentiation and inhibited the proliferation of neutrophil progenitors in BM to reduce neutrophil overproduction. These findings again emphasize the potential therapeutic benefits of CBP/P300 BRD inhibitors on neutrophil-enriched TNBC.

Besides TAN reduction, CBP/P300 BRD inhibition was demonstrated to stimulate the immune response. MHCI expression is downregulated in many cancer types to promote immune evasion, and low MHCI expression has been associated with poor response to ICB (50, 51, 69, 70). MHCI expression can be silenced epigenetically, and thus epigenetic inhibitors have been suggested as one potential treatment that might restore MHCI expression (69). CBP/P300 BRD inhibition induced MHCI expression in 2208L tumor cells in vivo, suggesting that CBP/P300 BRD might be involved in MHCI silencing. Moreover, an induced IFN response and elevated IFN-β expression were observed with CBP/P300 BRD inhibition in vivo. The IFN response and antigen presentation in tumor cells both correlate with a better CTL response and high levels of tumor-infiltrated lymphocytes that have been associated with better prognosis and treatment response in TNBC (71–73). CBP/P300 BRD inhibitors were reported previously to reduce differentiation and an immunosuppressive phenotype of Tregs in follicular lymphoma (74, 75). However, there is limited information about their effects on CTLs. One study suggested that CBP/P300 BRD inhibition may lead to a more activated phenotype in CTLs, but did not report an increase in CTL frequency in the CT26 tumors, a colon cancer and “hot tumor” model known to have high lymphocyte infiltration and respond to ICB (76). In contrast, neutrophil-enriched TNBC models are considered “cold tumors,” inferring minimal lymphocyte infiltration and complete resistance to ICB (7, 77). In this study, we report that CBP/P300 BRD inhibition increased infiltrated CTLs in a “cold tumor” model and demonstrated that CTLs played a significant role in tumor regression and persistent growth inhibition induced by the CBP/P300 BRD inhibitor. These results imply that CBP/P300 BRD inhibition has the potential to reprogram “cold tumors” to “hot tumors.”

Importantly, this study also tested the effects of the CBP/P300 BRD inhibitor in combination with chemotherapy and ICB. DTX was selected because it was shown previously to stimulate immune response in both TNBC mouse models and patients (78, 79). Taxanes and anti–PD-1 are a part of the standard-of-care regimen for early TNBC patients. In the current study, 2208L tumors exhibited a minimal response to DTX and anti–PD-1, but the addition of the CBP/P300 BRD inhibitor led to durable inhibition of tumor growth. Moreover, after 27 days of the treatment, we discontinued the CBP/P300 BRD inhibitor and DTX while continuing only anti–PD-1. The tumors in the combination treatment group displayed reduced tumor regrowth as compared with the single-agent-treated group. TANs returned to the tumors treated with the single agent, but reduced TANs were still observed in the end-stage tumors from the combination treatment group. CTLs activated by anti–PD-1 treatment were previously reported to induce apoptosis and inhibit the activity of MDSCs (80). Thus, we speculate that the combination treatment might lead to a more persistent reduction in immunosuppression and thus stronger T cell activation. These results suggest that the addition of a CBP/P300 BRD inhibitor may help improve the response of neutrophil-enriched TNBC to standard-of-care therapies and result in a durable immune response.

Lastly, we investigated the effects of CBP/P300 BRD inhibition on lung metastases since metastasis is the cause of mortality for the majority of TNBC patients (81). In primary tumors, CBP/P300 BRD inhibition was found to reduce the RNA expression of multiple genes associated with metastasis, implying that CBP/P300 BRD might play a role in metastasis. The 2208L model readily metastasizes to the lung, but always exhibits aggressive local dissemination and invasion, and therefore we could never fully resect the primary tumors to study spontaneous metastases. Accordingly, in this study, we used an experimental metastasis model to investigate the effects of CBP/P300 BRD inhibition on established lung metastases. CBP/P300 BRD inhibition significantly reduced the metastatic outgrowth of the 2208L model in the lung. Previous studies reported that neutrophils can support metastatic progression by inducing metastatic tumor cell proliferation and promoting angiogenesis (82). CBP/P300 BRD inhibition was found to decrease infiltrated neutrophils in 2208L lung metastases. Thus, CBP/P300 BRD inhibitors may also be effective in treating metastases of neutrophil-enriched TNBC.

We are aware that our study has several limitations. All the models used in this study were transplantable syngeneic mouse models, and therefore surgeries were required to implant the tumors. These surgeries might trigger local inflammation and thus transiently affect the TIME or the immune response systemically. Moreover, these models have relatively short latencies and rapid tumor growth, making it difficult to assess treatment effects during earlier stages of tumor progression and T cell responses, which may require long-term treatment studies. Ideally, spontaneous autochthonous models could be used, but their long latency and variability preclude their use for treatment studies that require large and matched cohorts of control and treatment groups. Differences between the mouse and human immune systems might also affect the TIME. Since the response to CBP/P300 BRD inhibition is T cell dependent, immunocompromised PDX models currently available are not suitable for these studies. To ensure that the responses observed were not dependent on the mouse strain, we selected models derived from both BALB/c and C57BL/6J backgrounds. Another limitation of the present study is that scRNA-seq does not infer changes in chromatin accessibility induced by CBP/P300 BRD inhibition. While single-cell ATAC sequencing coupled with scRNA-seq will provide more information such as changes in motif accessibility, due to the possibility of extracellular trap formation, neutrophils are routinely removed during sample preparation for single-cell ATAC sequencing.

In summary, this study suggests that CBP/P300 BRD inhibitors might provide therapeutic benefits to neutrophil-enriched TNBC by reducing immunosuppression and stimulating an antitumor immune response. With IACS-70654, a selective CBP/P300 BRD inhibitor, we determined that CBP/P300 BRD inhibition reduced the growth of neutrophil-enriched TNBC models. CBP/P300 BRD inhibition reduced TANs by reprogramming abnormal proliferation and differentiation of neutrophil progenitors in BM. CBP/P300 BRD inhibition also promoted the IFN response, induced MHCI expression in tumor cells, and increased infiltrated CTLs. These results also indicated that the CBP/P300 BRD inhibitor may improve the response of neutrophil-enriched TNBC to chemotherapy and ICB, and the combination treatment might elicit a more durable immune stimulation. While these preclinical studies will need to be validated in patients, they provide a rationale for future clinical trials in neutrophil-enriched cancers.

## Methods

### Sex as a biological variable

Our study exclusively examined female mice because TNBC is rare in men. Our study modeled TNBC in women only.

### Animal studies

All female WT BALB/c mice were purchased from Inotiv, and all female WT C57BL/6J mice were purchased from The Jackson Laboratory. All animals were housed in the Transgenic Mouse Facility at Baylor College of Medicine (BCM) with a 12-hour day/12-hour night cycle in climate-controlled conditions. All ethical regulations were complied with for all animal studies. The ethical endpoint for primary mammary tumor study is tumor size greater than 1500 mm^3^. The ethical endpoint for lung metastasis studies is a more than 20% decrease in body weight or a significant decrease in body condition.

#### Mammary tumor transplantation.

The generation of the *Trp53*-null tumor models has been described in previous publications (83, 84). All *Trp53*-null mammary tumor models were kept frozen as an established tumor bank. Before transplantation, frozen tumor pieces were thawed and cut into 1-mm chunks. Tumor transplantation was performed with 7- to 8-week-old WT BALB/c mice. One tumor chunk was implanted into the fourth mammary fat pad of each mouse. PyMT-N tumors were originally provided by Yang Gao from BCM. The frozen PyMT-N tumor pieces were first recovered and transplanted to the fourth mammary fat pad of a 7- to 8-week-old WT C57BL/6J mouse. When the tumor reached approximately 1 cm in diameter, it was harvested to obtain a freshly dissociated single-cell suspension, as described previously (85). Tumors were digested with 1 mg/mL collagenase type I (Sigma-Aldrich, 11088793001) and 1 μg/mL DNase (Sigma-Aldrich, 11284932001) for 2 hours at 37°C on a shaker. Short centrifugations were performed to enrich tumor cells. Then the pellets were trypsinized, counted, and filtered. PyMT-N tumor cells (250,000) were injected into the mammary fat pad of each mouse. For all studies, tumor growth was monitored by measuring the tumor volume using a digital caliper. All treatment studies started when the average tumor volume was more than 80 mm^3^. The mice were randomized into treatment groups by weight and tumor volume using RandoMice (86). Then, the weight and tumor volume measurements were performed 3 times a week.

#### Experimental lung metastasis model using tail vein injection.

To prepare for the tail vein injection, a 2208L tumor chunk was first implanted into the fourth mammary fat pad of a 7- to 8-week-old WT BALB/c mouse. When the tumor reached approximately 1 cm in diameter, the tumor was harvested and processed into a single-cell suspension, as described above. Lung metastases were generated via tail vein injection of 100,000 cells into each 6- 8-week-old female BALB/c mouse. Two mice were euthanized to collect the lung to ensure a successful generation of lung metastases before starting the treatment studies.

#### Treatment.

IACS-70654 was suspended in sterile 0.5% methylcellulose (Sigma-Aldrich, M0430) and administered orally at a dosage of 3.75 mg/kg on a 3-on/2-off schedule. The control group was given the same volume of 0.5% methylcellulose. For labeling proliferative cells, mice were injected i.p. with 60 mg/kg BrdU (Sigma-Aldrich, B-5002) 2 hours before euthanasia. Docetaxel (DTX) (LC Laboratories, D-1000) was dissolved in Tween 80 first and then diluted 1:4 with 16.25% ethanol. DTX was administered i.p. at 10 mg/kg weekly. RecombiMAb anti-mouse PD-1 (BioXCell, CP151) and RecombiMAb mouse IgG2a isotype (BioXCell, CP150) were administered i.p. at 200 μg per mouse every 3 days. InVivoMAb anti–mouse CD8α (BioXCell, BE0061) and InVivoMAb rat IgG2b isotype control (BioXCell, BE0090) were administered i.p. at 250 μg and 200 μg per mouse, respectively, every 4 days. All in vivo antibodies were diluted with InVivoPure pH 7.0 Dilution Buffer (BioXCell, IP0070). Mice were monitored for signs of toxicity at least 3 times per week.

### Flow cytometry

#### Tumor-infiltrated immune cells.

Tumors were processed and digested with collagenase as previously above. To enrich immune cells, the supernatants were collected after the short centrifugations. The supernatants were resuspended in RBC lysis buffer (BioLegend, 420301). The enriched cells were filtered, counted, and resuspended to 10 million cells/mL in FACS buffer (PBS + 2% FBS). Cells (1.0 × 10^6^) were first stained with Live/Dead Fixable Yellow (Thermo Fisher Scientific, L34968; 1:800). The cells were then blocked with anti–mouse CD16/CD32 antibody (BioLegend, 101320). Then, the cells were stained with antibodies for cell surface markers from each panel. For intracellular staining, the cells were fixed and permeabilized using the FOXP3/Transcription Factor Staining Buffer Set (Thermo Fisher Scientific, 00-5523-00). The cells were then blocked with 2% rat serum (Sigma-Aldrich, R9759) and 2% goat serum (Sigma-Aldrich, G9023) in the permeabilization buffer from the buffer set. The cells were then stained with antibodies for intracellular markers from each panel.

#### Blood and BM myeloid cells.

For PBMCs, blood was collected retro-orbitally, and 30 μL of blood from each mouse was incubated in RBC lysis buffer for 30 minutes. For BM, the mice were injected i.p. with 60 mg/kg BrdU 24 hours before euthanasia. Then, BM cells were extracted from 1 femur bone of each mouse. The cells were incubated in RBC lysis buffer for 2 minutes and filtered. BM cells were stained with Live/Dead Fixable Yellow at 1:800 dilution and blocked with anti–mouse CD16/CD32 antibody. The cells were next incubated with antibodies. Then, PBMCs were washed and stained with NucBlue Live ReadyProbes Reagent (Thermo Fisher Scientific, R37605) in PBS for data acquisition. BM cells were fixed and permeabilized using the FOXP3/Transcription Factor Staining Buffer Set overnight. On the next day, BM cells were washed with permeabilization buffer and incubated in 0.3 mg/mL DNase I at 37°C for 1 hour. Then, BM cells were stained with BrdU PE (BioLegend, 339812; 1:100) for 30 minutes.

After staining, all cells were resuspended in PBS for acquisition on the Attune NxT flow cytometer at the BCM FACS and Cell Sorting core. The collected data were compensated and analyzed using FlowJo v10 software (FlowJo, RRID: SCR_008520). Detailed information about the antibodies used for each flow cytometry panel is provided in Supplemental Table 30.

### Histology

Primary tumor and lung tissues were fixed in 4% paraformaldehyde overnight. The fixed tissues were embedded in paraffin and sectioned at 5 μm at the Breast Center Pathology Core at BCM. Serial sectioning of lung metastases was performed by collecting 4–6 5-μm sections every 150 μm.

#### IHC staining.

The tissue sections were first deparaffinized and rehydrated. Antigen retrieval was performed by immersing tissue sections in Tris-EDTA antigen retrieval buffer (10 mM Tris, 1 mM EDTA, 0.05% Tween 20, pH 9.0) on a steamer for 30 minutes. Endogenous peroxidases were blocked by immersing tissue sections in 3% hydrogen peroxide (Thermo Fisher Scientific, H323-500; diluted 1:10 in PBS) for 10 minutes. The sections were then incubated for 1 hour in the IHC blocking buffer containing 3% BSA (Sigma-Aldrich, A7906) and 5% goat serum (Sigma-Aldrich, G9023) in PBS. Next, tissue sections were incubated with primary antibodies at 4°C overnight. The primary antibodies used include anti-S100A8 (R&D Systems, MAB3059; 1:5000), anti-F4/80 (Cell Signaling Technology, 70076S; 1:500), anti-BrdU (Abcam, ab6326; 1:1000) and anti-CD8α (Cell Signaling Technology, 98941S; 1:500). The sections were then incubated in biotin-conjugated anti-rat (Vector Laboratories, PI-9400-1) or anti-rabbit (Vector Laboratories, PI-1000-1) secondary antibodies at 1:1000 dilution for 1 hour at room temperature. Next, sections were incubated with VECTASTAIN Elite ABC HRP Reagent (Vector Laboratories, PK7100) for 30 minutes and treated with ImmPACT DAB peroxidase substrate (Vector Laboratories, sk-4105) until optimal signals were observed. The slides were counterstained with Harris Hematoxylin with Glacial Acetic Acid (Poly Scientific, S212A).

#### H&E staining.

After deparaffinization and rehydration, the slides were stained with Harris Hematoxylin with Glacial Acetic Acid and Eosin Phloxine Alcoholic Working Solution (Poly Scientific, S176).

#### Imaging and quantification.

The slides are dehydrated and mounted in Poly-Mount Xylene (Poly Scientific, 24176–120). Images of all slides were acquired using the Olympus BX40 light microscope and MPX-5C pro low-light camera at ×20 magnification or with Aperio ImageScope (Leica Biosystems). Quantification was performed by Fiji (87).

### scRNA-seq

#### Single-cell suspension preparation.

For all scRNA-seq experiments, each sample consisted of cells pooled from 3 mice. Mice were euthanized 24 hours after the last treatment. Tumors were processed and digested as described above. Dissociated cells were stained with Ghost Dye UV 450 (Tonbo Biosciences, 13-0868) for 10 minutes on ice and sorted by FACS to select viable cells. BM cells were harvested from the tibia and femur bones using FACS buffer and then passed through a 70-μm strainer. Following centrifugation, the cells were resuspended in red blood cell lysis buffer (Tonbo Biosciences, TNB-4300-L100). Total BM cells were stained with CD45 APC (Tonbo Biosciences, 20-0451-U100), Ter119 PE (BioLegend,116207), and DAPI (Invitrogen, R37606) at 4°C for 15 minutes, followed by FACS of BM DAPI^–^CD45^+^Ter119^–^ cells. To isolate BM HSPCs, total BM cells were processed as above and then stained with anti-mouse biotinylated lineage antibodies (CD11b/Gr-1/B220/Ter119/CD3e) (BD Bioscience, 559971), followed by staining with Streptavidin APC (Tonbo Biosciences, 20-4317), CD45 VF450 (Tonbo Biosciences, 75-0451), c-Kit PE/Cy7 (Tonbo Biosciences, 60-1172), and DAPI. The DAPI^–^CD45^+^Lin^–^c-Kit^+^ (HSPCs) were then sorted by FACS for analysis. The single-cell suspensions from tumor or BM were then tagged with CellPlex barcoding oligo (10× Genomics, 1000261), and immediately delivered to the Single Cell Genomics Core at BCM for scRNA-seq library preparation.

#### Library preparation and sequencing.

The single-cell gene expression library was prepared according to the Chromium Single Cell Gene Expression 3′ v3.1 kit along with the feature barcoding kit (10× Genomics, 1000262). After passing quality control, the next-generation sequencing of libraries was performed on NovaSeq 6000 (Illumina).

#### Preprocessing of scRNA-seq datasets.

Upon receiving the raw sequencing data, we prepared the CellRanger multi (v7.2.0) pipeline to conduct alignment, read counts, and sample demultiplexing. For the tumor dataset, we adjusted the barcode assignment confidence to 0.8 to include more cells for downstream analysis. Downstream analyses of scRNA-seq were performed using the Seurat (v4.4.0) package in R (R version 4.3.1; https://github.com/satijalab/seurat). For quality control, cells with more than 200 and less than 6000 read counts were kept. Cells with more than 1000 UMI counts and greater than 10% mitochondrial ratio were removed. Datasets were downsampled to the lowest cell number in the group and normalized. The samples were integrated using the IntegrateData function.

#### Clustering and cluster annotation.

The integrated data were used to perform principal component analysis. Then, the first 30 principal components identified were used for uniform manifold approximation and projection (UMAP) analysis. The clusters were annotated using SingleR Immgen (https://github.com/SingleR-inc/celldex) and then verified manually. The macrophage and monocyte populations were isolated and re-clustered at a resolution of 0.4. The CD45^+^ BM cells were clustered first annotated using SingleR Immgen. The neutrophils were isolated and re-clustered at a resolution of 0.8. Clusters of neutrophils were annotated manually based on published markers (26, 30, 31). For HSPCs, all cells were clustered, and non-HSPC clusters were excluded based on published markers (9). The HSPCs were re-clustered at a resolution of 1 and re-annotated using published markers (9). All pseudotime plots were generated using Monocle 3 (https://github.com/cole-trapnell-lab/monocle3). The cell cycle scoring was performed using the CellCycleScoring function in Seurat. The gene signature expression scoring was performed using the AddModuleScore_Ucell function from the UCell package (https://github.com/carmonalab/UCell). The IFN neutrophil (33) and neutrophil differentiation signatures (43) were retrieved from published studies.

#### Differentially expressed genes.

Differentially expressed genes were identified with FindMarkers function from Seurat using the MAST test between 2 groups. The volcano plot was generated using the EnhancedVolcano package. The clusterProfiler package was used for GO pathway enrichment analyses.

### Clinical data analysis

The original data were from the I-SPY2 trial (50), and the processed data from paclitaxel and pembrolizumab-treated patients were retrieved from ClinicalOmicsDB (51). The violin plots of individual gene expressions were generated in R. The statistics and the plot of KEGG pathway expression were generated at ClinicalOmicsDB.

### Cytokine profiling

Snap-frozen tumor chunks were homogenized with T-PER Tissue Protein Extraction Reagent (Thermo Fisher Scientific, 78510) containing cOmplete, EDTA-free Protease Inhibitor Cocktail with zirconium beads in BeadBlaster 24 Microtube Homogenizer. Protein concentrations were measured with the BCA Protein Assay Kit, and homogenates were diluted to 1.8 μg/μL. Plasma samples were diluted 2-fold with PBS. All samples were assayed using Mouse Cytokine/Chemokine 44-Plex Discovery Assay Array (MD44) at Eve Technologies Corp.

### Statistics

For comparison between the 2 groups, an unpaired, 2-tailed Student’s *t* test was used. For comparison among 3 or more groups and pairwise comparisons, ordinary 1-way ANOVA and Tukey’s multiple-comparison test were used. Two-way ANOVA and Šidák’s multiple-comparison test were used for analyzing tumor volume changes, log(fold changes) of tumor volume, or body weight changes over time. For Kaplan-Meier curves, the log-rank test was performed, and for multiple comparisons, Bonferroni’s method was used to adjust the *P* value. A *P* value of less than 0.05 was considered significant. All statistical analyses mentioned above were performed with GraphPad Prism 9. For scRNA-seq, statistical analyses were performed in R. Log_2_(fold change) greater than 0.5 or less than –0.05, and an adjusted *P* value of less than 0.01 was considered significant in differential gene expression analyses. An adjusted *P* value of less than 0.05 was considered significant in GO pathway enrichment analyses. For the clinical data, an absolute value of meta *P* of less than 0.05 was considered significant.

### Study approval

The animal experiments were performed at BCM following a protocol (AN-504) approved by the IACUC.

### Data availability

All scRNA-seq data have been submitted to the NCBI GEO database and are publicly available (GSE264627). All data points shown in graphs are provided in the Supporting Data Values file. All data and experimental information from this study are in the article or the supplemental material and available from the corresponding author upon request.

## Author contributions

XY designed research studies, conducted experiments, acquired data, analyzed data, and wrote the manuscript. XH and HLC designed and conducted scRNA-seq experiments. NZ, DAP, AJS, SJC, and NL conducted experiments and acquired data. FL processed scRNA-seq data. KL, MJS, and PJ provided IACS-70654 and its pharmacological characterization. XHFZ provided resources for scRNA-seq experiments. JMR designed and supervised the research study and edited the manuscript.

## Supplementary Material

Supplemental data

Unedited blot and gel images

Supplemental tables 3-12

Supplemental tables 13-20

Supplemental tables 21-28

Supplemental table 29

Supplemental table 30

Supporting data values

## Version 1 09/17/2024

In-Press Preview

## Version 2 10/22/2024

Electronic publication
