# Supplementary material for: CREB-binding protein/P300 bromodomain inhibition reduces neutrophil accumulation and activates antitumor immunity in triple-negative breast cancer
Source: JCI Insight. 2024 Sep 17;9(20):e182621. doi: 10.1172/jci.insight.182621 (PMC11533985; doi:10.1172/jci.insight.182621)
Supplement: Supplemental data [file jciinsight-9-182621-s008.pdf]

Supplemental Methods

IACS-70654 pharmacological characterization

Specific binding of the CBP or BRD4 bromodomain to the acetylated peptide derived from the H4 histone substrate (tetra acetylated H4 (1-21) Ac-K5/8/12/16) was measured in the absence or presence of inhibitors. The GST-tagged bromodomains of CBP (1081-1197) and BRD4 (49-170) were obtained from BPS Bioscience and binding to the biotinylated H4 (1-21) Ac-K5/8/12/16 (AnaSpec. 64989) was assessed via AlphaScreen technology (Perkin Elmer). For CBP AlphaScreen assay, 5 nM GST-CBP (1081- 1197) and 20 nM biotin-H4 (1-21) Ac-K5/8/12/16 (AnaSpec. 64989) were incubated with varying concentrations of CBP inhibitors in 15 µl of buffer containing 50 mM HEPES 7.5, 100 nM NaCl, 1 mM TCEP, and 0.003% Tween-20. After 30 minutes of incubation at room temperature, 15 µl of detection buffer (BPS Bio. 33006) containing 7 mg/ml of Glutathione AlphaLisa acceptor beads (Perkin Elmer AL109) and 14 µg/ml of Streptavidin donor beads (Perkin Elmer 676002) was then added to the previous mixture. The reaction was incubated for an additional 2 h at room temperature, and the AlphaScreen signal was quantified using the Envision Multilabel plate reader. As a negative control, GST-CBP (1081-1197) was incubated with the non-acetylated biotin-H4 (1-21) peptide (AnaSpec. 62555) and in the presence of 0.25% of final DMSO concentration. For the BRD4 AlphaScreen assay, the binding of 2.5 nM of BRD4 (49-170) to 10 nM biotin-H4 (1-21) Ac-K5/8/12/16 (AnaSpec. 64989) was assessed following the same procedure described for the CBP assay. The standard dose response curves were fitted by Genedata Screener software using the variable-slope model. Only Signal and Dose in the equation were treated as known values. Screening of IACS-70654 against 32 bromodomain proteins was performed by BromoMAX (DiscoverX). To measure Kds, BromoKdELECT was used (DiscoverX).

In vitro culture of *Trp53*-null tumor cells

T12 tumor cells were cultured in DMEM/Ham's F-12 medium (GenDEPOT, CM017-050) with 10% fetal bovine serum (GenDEPOT, F0900-050), 5 μg/ml insulin (Millipore Sigma, I-5500), 1 μg/ml hydrocortisone (Millipore Sigma, H0888), 10 ng/ml epidermal growth factor (Sigma, SRP3196), and 1X Antibiotic-Antimycotic (ThermoFisher Scientific, 15240062). 2208L tumor cells were cultured in DMEM medium (GenDEPOT, CM002-050) with 10% fetal bovine serum, and 1X Antibiotic-Antimycotic. IACS-70654 was dissolved in DMSO as 1mM stock and further diluted with cell medium for treatment in vitro.

Immunoblotting

Tumor cells cultured in vitro were mixed with and lysed by tissue lysis buffer (62.5 mM Tris-HCl pH 6.8, 2% SDS) containing cOmplete, EDTA-free Protease Inhibitor Cocktail (Sigma-Aldrich, 11873580001). Tumors were snap-frozen and later homogenized in tissue lysis buffer with zirconium beads (Benchmark Scientific, D1132-30,) in BeadBlaster 24 Microtube Homogenizer. The cell/tumor lysates were heated at 98°C for 8 minutes. Protein concentrations were measured with the BCA Protein Assay Kit (ThermoFisher, 23227). The protein extracts were loaded on an SDS-PAGE system and then transferred to polyvinylidene difluoride membranes (Millipore, IPVH00010). The primary antibodies used include CBP (Cell Signaling, 7389, 1:1000), P300 (Cell Signaling, 57625, 1:1000), acetyl-histone H3 (Lys27) (Cell Signaling, 8173, 1:1000), acetyl-histone H3 (Lys9) (Cell Signaling Technology, 9649, 1:1000), histone H3 (Cell Signaling Technology, 4499, 1:2000), GAPDH (Cell Signaling Technology, 2118, 1:2000), FGFR1 (Cell Signaling Technology, 9740, 1:1000) and β-Actin (Cell Signaling Technology, 3700, 1:5000).

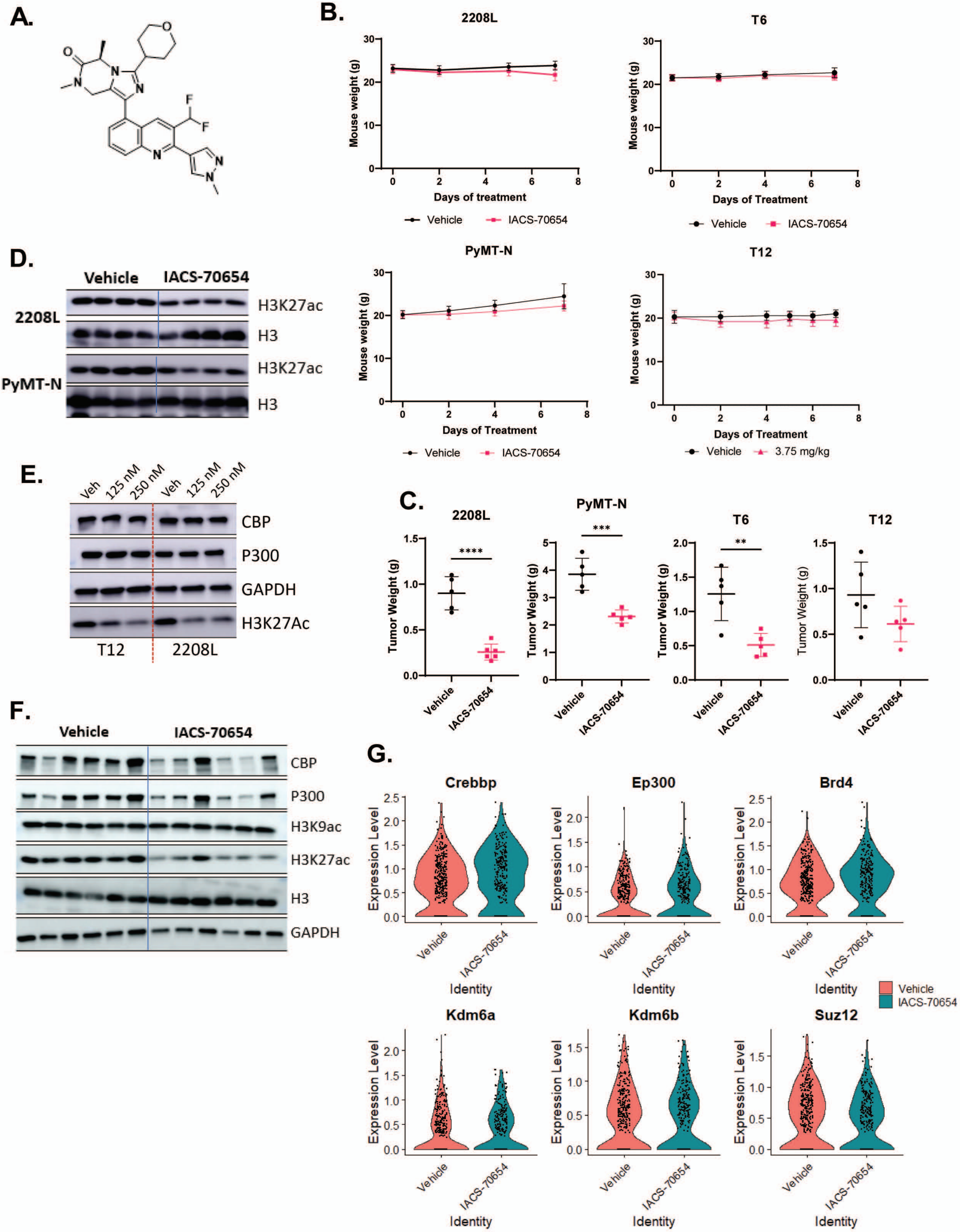

A.
B.
2208L
T6
Mouse weight (g)
Days of treatment
Days of Treatment
Vehicle
IACS-70654
D.
Vehicle
IACS-70654
2208L
PyMT-N
H3K27ac
H3
PyMT-N
T12
3.75 mg/kg
E.
Veh
125 nM
250 nM
CBP
P300
GAPDH
H3K27Ac
T12
2208L
C.
Tumor Weight (g)
****
***
**
F.
H3K9ac
G.
Crebbp
Ep300
Brd4
Kdm6a
Kdm6b
Suz12
Expression Level
Identity

Supplemental Figure S1. Structure of IACS-70654 and the weights of mice and 2208L tumors treated with IACS-70654 for 7 days. **A.** Chemical structure of IACS-70654. **B.** Mouse weights of the *Trp53*-null (2208L, T6, and T12) and PyMT-N tumor-bearing mice during the 7-day treatment of vehicle or IACS-70654. For 2208L, n = 6, and for all other models, n = 5. Error bars represent SD. **C.** Weight of 2208L, PyMT-N, T6, and T12 tumors after 7-day treatment of vehicle or IACS-70654. For all models, n = 5 for each treatment arm. Two-tailed unpaired Student's t test was used. **, $p<0.01$; ***, $p<0.001$; ****, $p<0.0001$. Error bars represent SD. **D.** Immunoblot analyses of Histone H3K27 acetylation (H3K27ac), the target biomarker for IACS-70654, in 2208L and PyMT-N tumors. Four biological replicates were used per group. Histone H3 was used as the loading control. **E.** Immunoblot analysis of CBP, P300, and H3K27ac in T12 and 2208L cells treated with two different concentrations of IACS-70654 in vitro for 4 h. GAPDH was used as the loading control. **F.** Immunoblot analysis of CBP, P300, H3K9ac, and H3K27ac in 2208L tumors treated with vehicle or IACS-70654. Histone H3 and GAPDH were used as loading controls. Six biological replicates were used for each treatment arm. **G.** Violin plots showing RNA expression of various epigenetic factors (*Crebbp, Ep300, Brd4, Kdm6a, Kdm6b and Suz12*) from single-cell RNA sequencing (scRNA-seq) analyses of tumors cells in 2208L tumors treated with vehicle or IACS-70654.

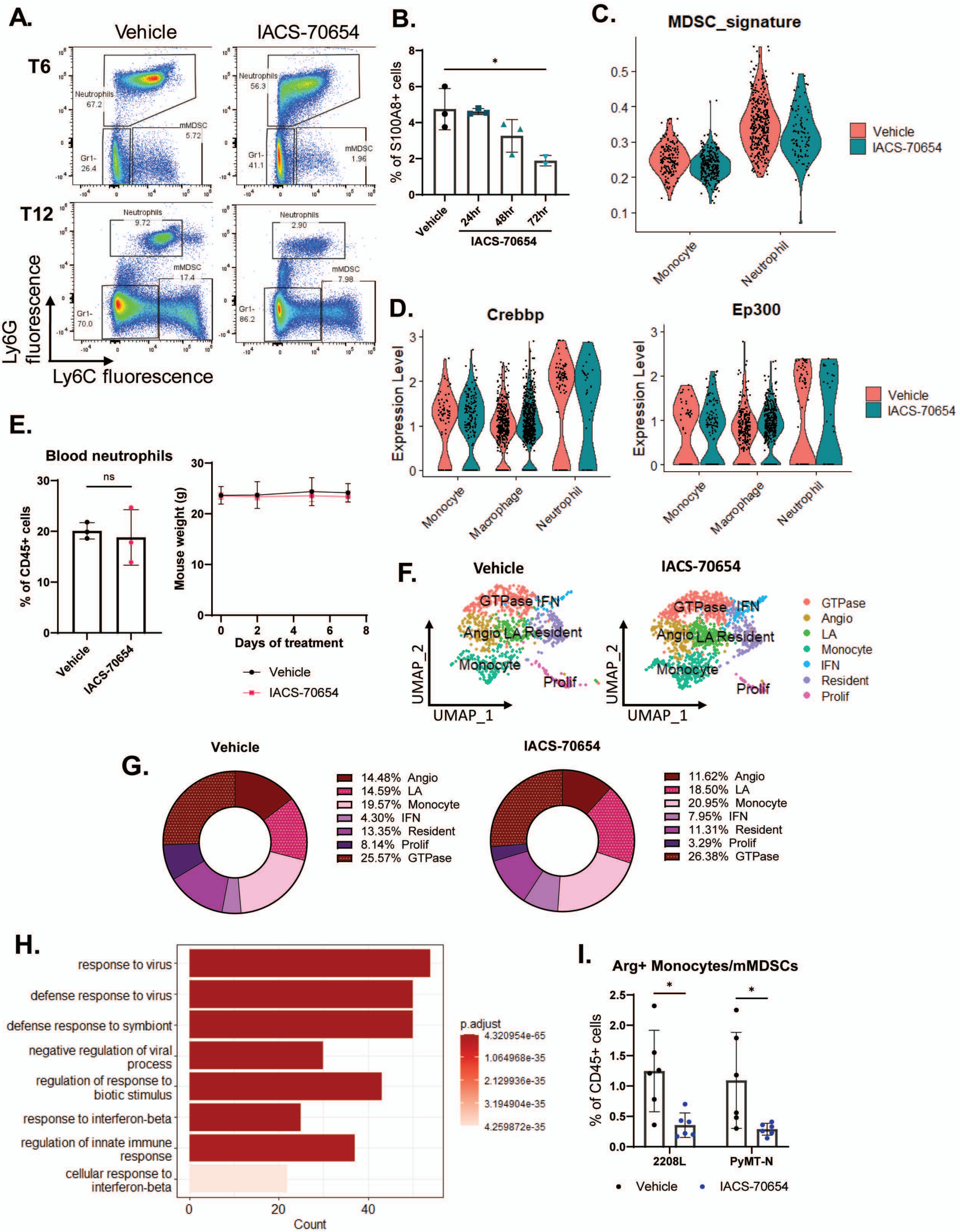
A.
Vehicle
IACS-70654
T6
T12
Neutrophils
mMDSC
Gr1-
Ly6G fluorescence
Ly6C fluorescence
B.
% of S100A8+ cells
Vehicle
24hr
48hr
72hr
IACS-70654
C.
MDSC_signature
Monocyte
Neutrophil
Vehicle
IACS-70654
D.
Crebbp
Ep300
Expression Level
Monocyte
Macrophage
Neutrophil
E.
Blood neutrophils
ns
% of CD45+ cells
Mouse weight (g)
Days of treatment
Vehicle
IACS-70654
F.
Vehicle
IACS-70654
GTPase
Angio
LA
Monocyte
IFN
Resident
Prolif
UMAP_1
UMAP_2
G.
Vehicle
14.48% Angio
14.59% LA
19.57% Monocyte
4.30% IFN
13.35% Resident
8.14% Prolif
25.57% GTPase
IACS-70654
11.62% Angio
18.50% LA
20.95% Monocyte
7.95% IFN
11.31% Resident
3.29% Prolif
26.38% GTPase
H.
response to virus
defense response to virus
defense response to symbiont
negative regulation of viral process
regulation of response to biotic stimulus
response to interferon-beta
regulation of innate immune response
cellular response to interferon-beta
p.adjust
4.320954e-65
1.064968e-35
2.129936e-35
3.194904e-35
4.259872e-35
Count
I.
Arg+ Monocytes/mMDSCs
% of CD45+ cells
2208L
PyMT-N
Vehicle
IACS-70654

Supplemental Figure S2. Changes in tumor-associated myeloid cells after IACS-70654 treatment. **A.** Representative density plots of Ly6G versus Ly6C showing gating strategy for and changes in myeloid populations of T6 and T12 tumors. The gating was performed on $CD45^{+}CD11b^{+}$ populations. **B.** Quantification of S100A8 staining on sections of 2208L tumors treated with vehicle or IACS-70654 (24, 28, or 72-hour treatment). Three representative images were analyzed for each tumor, and three biological replicates were used for each treatment arm. Two-tailed unpaired Student's *t* test was used. *, $p<0.05$. Error bars represent SD. **C.** Violin plots showing expression of the MDSC signature in tumor-associated monocytes and neutrophils in 2208L tumors treated with vehicle or IACS-70654. **D.** Violin plots showing RNA expression of *Crebbp* and *Ep300* from scRNA-seq analyses of tumor-associated neutrophils, macrophage and monocytes of 2208L tumors treated with vehicle or IACS-70654. **E.** Non-tumor-bearing WT BALB/c mice treated with vehicle or IACS-70654 for 7 days. Left: Flow cytometry analyses of blood neutrophils. Two-tailed unpaired Student's *t* test was used. Three biological replicates were used for each treatment arm. ns, $p>0.05$. Error bars represent SD. Right: Changes in mouse weight over treatment. Error bars represent SD. **F.** UMAP of TAM subpopulations with annotations in 2208L tumors treated with vehicle or IACS-70654. TAM subtypes include High GTPase expressing (GTPase), IFN response gene expressing (IFN), proangiogenic (Angio), lipid-associated (LA), resident, and proliferating (Prolif) TAMs. **G.** The fractions of TAM subtypes in TAMs of 2208L tumors treated with vehicle or IACS-70654 derived from scRNA-seq analyses. **H.** GO pathway enrichment analysis of the enriched genes (Log2 fold change > 0.5 and adjusted p-value < 0.01) in integrated IFN TAMs of 2208L tumors. Biological Process gene sets from the GO database were used. The top 8 pathways were listed with numbers of genes enriched. **I.** Quantification of Arginase 1 (Arg)-positive tumor-infiltrated monocytes/mMDSCs as percentages of $CD45^{+}$ cells in 2208L and PyMT-N models treated with vehicle or IACS-70654 using flow cytometry. Monocytes are defined by $Ly6G^{-}Ly6C^{+}$. Two-tailed

unpaired Student's *t* test was used. *, $p<0.05$. For both models, $n \geqslant 5$ for each treatment arm.

Error bars represent SD.

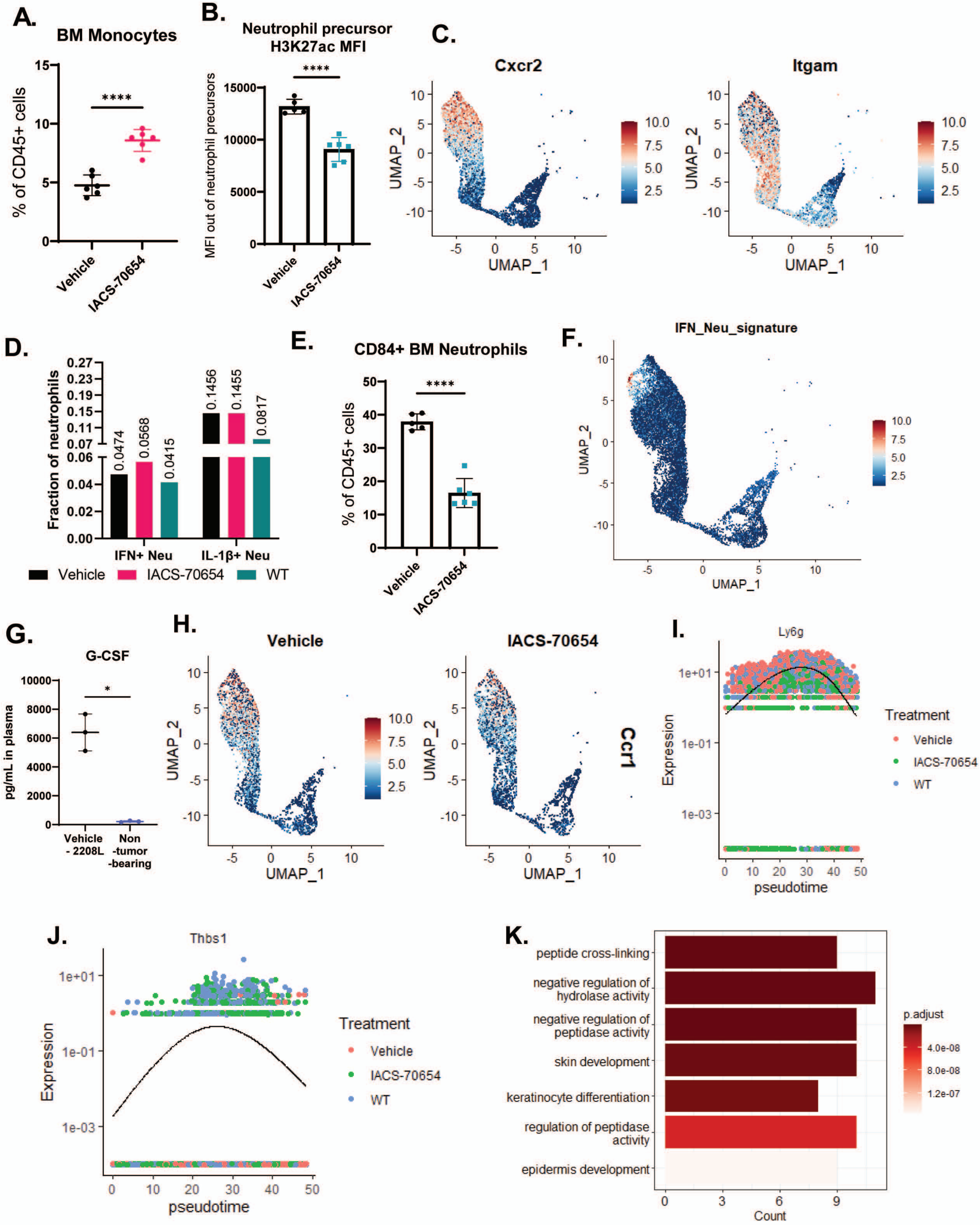

A.
BM Monocytes
% of CD45+ cells
****
Vehicle
IACS-70654
B.
Neutrophil precursor
H3K27ac MFI
MFI out of neutrophil precursors
****
Vehicle
IACS-70654
C.
Cxcr2
Itgam
UMAP_1
UMAP_2
D.
Fraction of neutrophils
0.0474
0.0568
0.0415
0.1456
0.1455
0.0817
IFN+ Neu
IL-1β+ Neu
Vehicle
IACS-70654
WT
E.
CD84+ BM Neutrophils
% of CD45+ cells
****
Vehicle
IACS-70654
F.
IFN_Neu_signature
UMAP_1
UMAP_2
G.
G-CSF
pg/mL in plasma
*
Vehicle - 2208L
Non -tumor -bearing
H.
Vehicle
IACS-70654
Ccr1
UMAP_1
UMAP_2
I.
Ly6g
Expression
pseudotime
Treatment
Vehicle
IACS-70654
WT
J.
Thbs1
Expression
pseudotime
Treatment
Vehicle
IACS-70654
WT
K.
peptide cross-linking
negative regulation of hydrolase activity
negative regulation of peptidase activity
skin development
keratinocyte differentiation
regulation of peptidase activity
epidermis development
p.adjust
4.0e-08
8.0e-08
1.2e-07
Count

Supplemental Figure S3. ScRNA-seq analyses of BM neutrophils. **A.** Quantification of monocytes as percentages of $CD45^+$ cells in the BM of 2208L tumor-bearing mice treated vehicle or IACS-70654 and non-tumor-bearing mice using flow cytometry. Monocytes are defined by $CD11b^+Ly6G^-Ly6C^+$. Two-tailed unpaired Student's *t* test was used. Error bars represent SD. ****, p<0.0001. Six biological replicates were used per group. **B.** MFI of H3K27ac in flow cytometry analyses of neutrophil precursors ($CD11b^+Ly6G^{low/med}F4/80^-$) in 2208L tumors treated with vehicle or IACS-70654. For each group, n ⩾ 5. Error bars represent SD. Two-tailed unpaired Student's t test was used. ****, p<0.0001. **C.** Expression distribution of *Cxcr2* and *Itgam* in BM neutrophils. **D.** Fraction of IFN+ and IL-1β neutrophils in BM neutrophils of 2208L tumor-bearing mice (treated with vehicle or IACS-70654) and non-tumor-bearing mice. The fraction values are labeled. **E.** Flow cytometry analysis of CD84-positive BM neutrophils in 2208L tumor-bearing mice treated with vehicle or IACS-70654. Two-tailed unpaired Student's *t* test was used, and *n* ⩾ 5. ****, p<0.0001. **F.** Expression distribution of IFN-stimulated neutrophil signature in integrated BM neutrophils. **G.** Quantification of G-CSF level in plasma from 2208L tumor-bearing and non-tumor-bearing mice by the cytokine/chemokine array. **H.** Expression distribution of *Ccr1* in BM neutrophils of 2208L tumor-bearing mice treated with vehicle or IACS-70654. **I.** RNA expression of *Ly6g* versus pseudotime in BM neutrophils. **J.** RNA expression of *Thbs1* versus pseudotime in BM neutrophils. **K.** GO pathway enrichment analysis of the significantly upregulated genes (Log_2 fold change > 0.5 and adjusted p-value < 0.01) in mature BM neutrophils of 2208L tumor-bearing mice treated with IACS-70654 compared to those treated with vehicle. Biological Process (BP) gene sets from the GO database were used. The top 7 terms were listed with numbers of genes enriched.

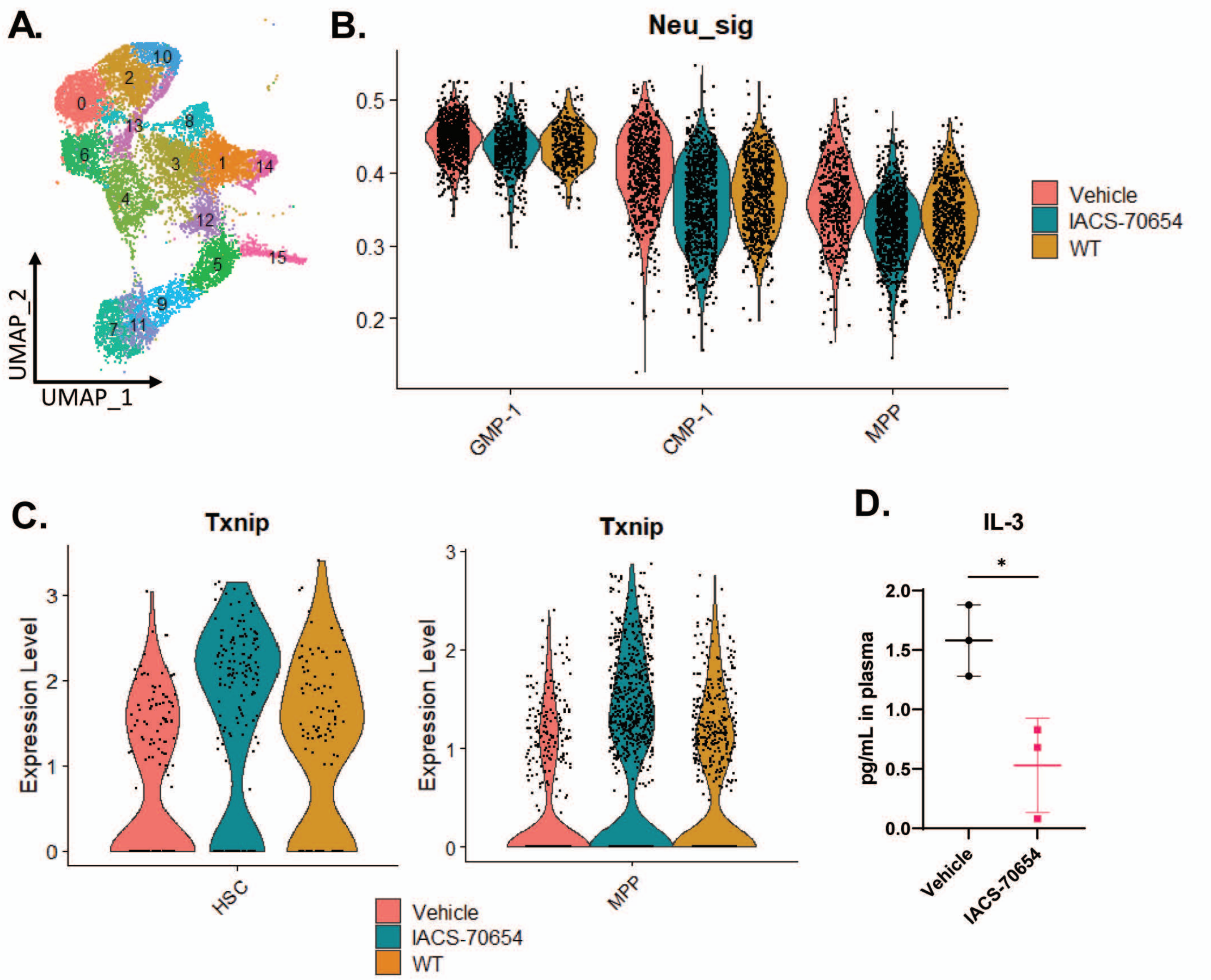
A.
B.
Neu_sig
0.5
0.4
0.3
0.2
GMP-1
CMP-1
MPP
Vehicle
IACS-70654
WT
UMAP_2
UMAP_1
C.
Txnip
Expression Level
HSC
MPP
D.
IL-3
pg/mL in plasma
2.0
1.5
1.0
0.5
0.0
*
Vehicle
IACS-70654

Supplemental Figure S4. ScRNA-seq analyses of HSPCs. **A.** UMAP plot of integrated HSPCs without annotation. **B.** Violin plot showing expression of neutrophil differentiation signature in GMP-1, CMP-1, and MPP in 2208L tumor-bearing mice (treated with vehicle or IACS-70654) or non-tumor-bearing (WT) mice. **C.** Violin plots showing the RNA expression of *Txnip* in HSCs and MPPs of 2208L tumor-bearing mice treated with vehicle or IACS-70654 and non-tumor-bearing WT mice. **D.** Quantification of IL-3 level in plasma collected from 2208L tumor-bearing mice treated with vehicle or IACS-70654 by the cytokine/chemokine array. Three biological replicates were used for each treatment arm. Two-tailed unpaired Student's *t* test was used. Error bars represent SD. *, $p<0.05$. Error bars represent SD.

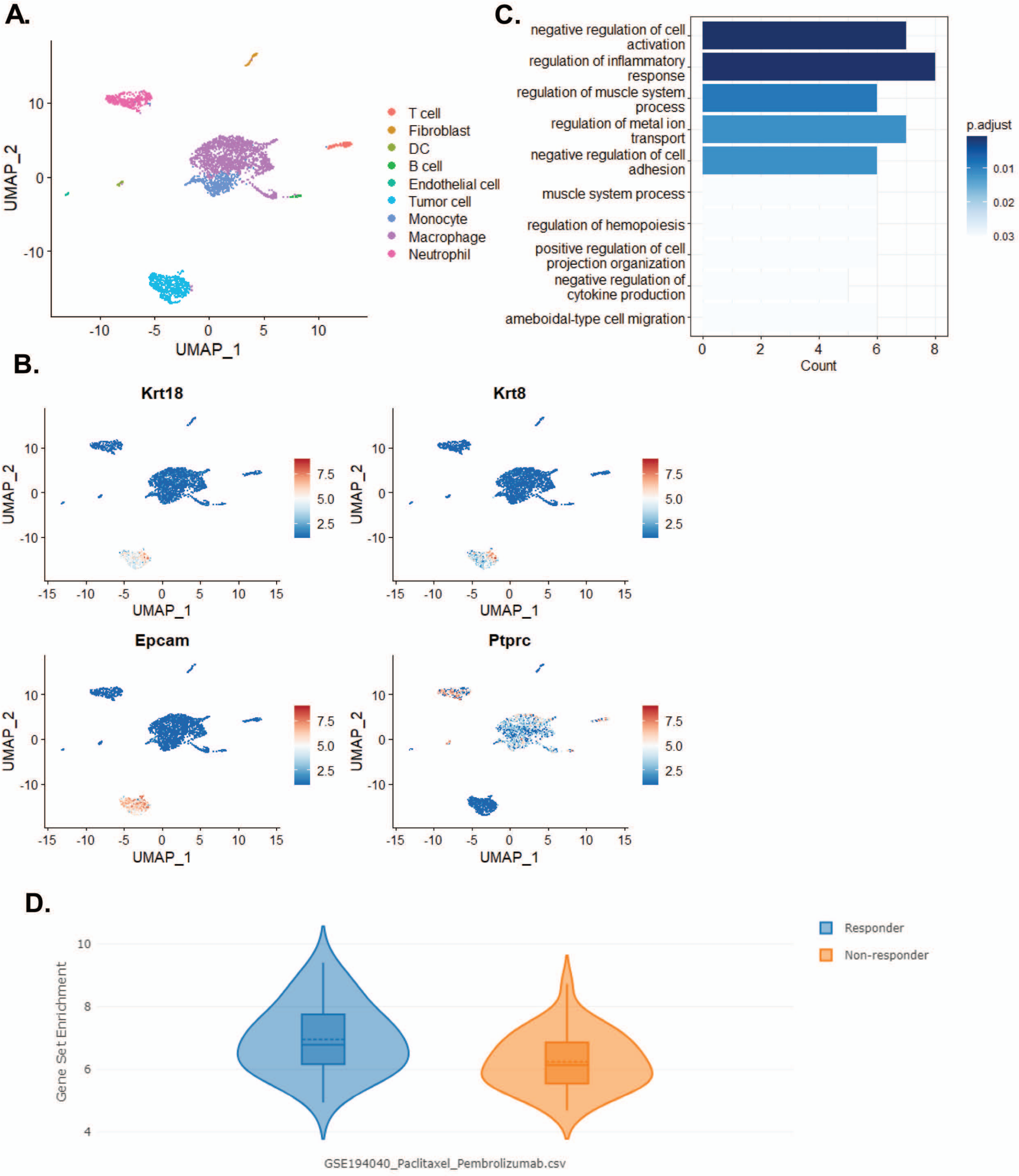
A.
UMAP_2
UMAP_1
T cell
Fibroblast
DC
B cell
Endothelial cell
Tumor cell
Monocyte
Macrophage
Neutrophil
B.
Krt18
Krt8
Epcam
Ptprc
C.
negative regulation of cell activation
regulation of inflammatory response
regulation of muscle system process
regulation of metal ion transport
negative regulation of cell adhesion
muscle system process
regulation of hemopoiesis
positive regulation of cell projection organization
negative regulation of cytokine production
ameboidal-type cell migration
Count
p.adjust
D.
Gene Set Enrichment
Responder
Non-responder
GSE194040_Paclitaxel_Pembrolizumab.csv

Supplemental Figure S5. ScRNA-seq analyses of 2208L tumors treated with vehicle or IACS-70654. **A.** UMAP of integrated 2208L tumor samples treated with vehicle and IACS-70654 with cell type annotations. **B.** Expression distribution of *Krt18, Krt8, Epcam,* and *Ptprc* in integrated 2208L tumor samples. **C.** GO pathway enrichment analysis of the significantly downregulated genes (Log_2 fold change < -0.5 and adjusted p-value < 0.01) in 2208L tumor cells treated with IACS-70654 compared to those treated with vehicle. Biological Process (BP) gene sets from the GO database were used. The top 10 terms were listed with numbers of genes enriched. **D.** RNA expression of KEGG antigen processing and presentation pathway in human TNBC patients who did (responders) or did not (non-responders) achieve pathological complete response (pCR) after treatment of paclitaxel and pembrolizumab (anti-PD1). The graph was generated at ClinicalOmicsDB (1).

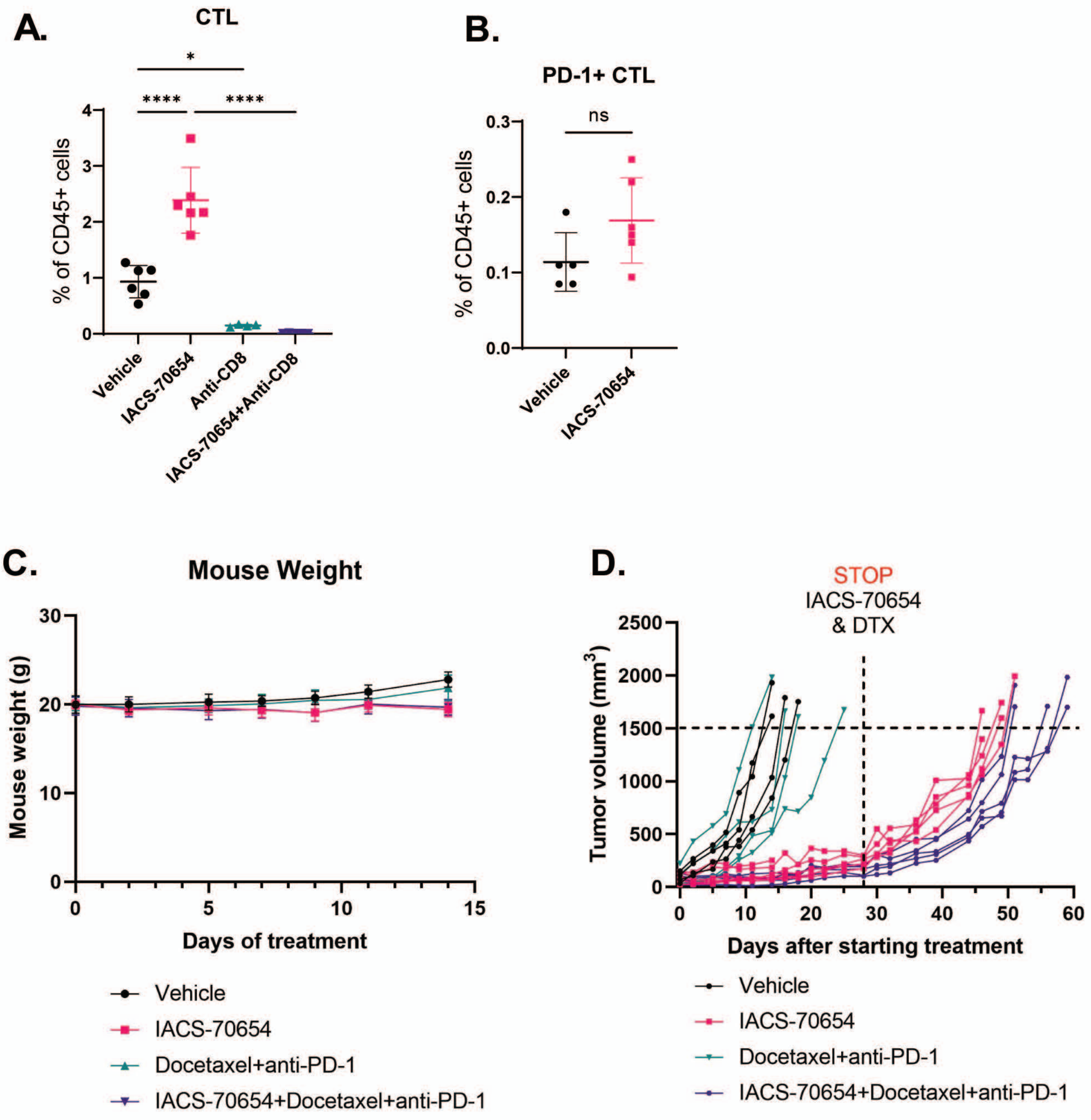

A.
CTL
*
****
****
% of CD45+ cells
0
1
2
3
4
Vehicle
IACS-70654
Anti-CD8
IACS-70654+Anti-CD8
B.
PD-1+ CTL
ns
% of CD45+ cells
0.0
0.1
0.2
0.3
Vehicle
IACS-70654
C.
Mouse Weight
Mouse weight (g)
0
10
20
30
0
5
10
15
Days of treatment
Vehicle
IACS-70654
Docetaxel+anti-PD-1
IACS-70654+Docetaxel+anti-PD-1
D.
STOP
IACS-70654
& DTX
Tumor volume (mm3)
0
500
1000
1500
2000
2500
0
10
20
30
40
50
60
Days after starting treatment
Vehicle
IACS-70654
Docetaxel+anti-PD-1
IACS-70654+Docetaxel+anti-PD-1

Supplemental Figure S6. Combination treatment of IACS-70654 with DTX and anti-PD-1 and anti-CD8. **A.** Quantification of CTLs as percentages of CD45+ cells in 2208L tumors treated vehicle or IACS-70654 with or without CTL depletion using flow cytometry. Ordinary one-way ANOVA and Tukey's multiple comparisons test were used. For all groups, more than four biological replicates were used. ****, $p<0.0001$; *, $p<0.05$. Error bars represent SD. **B.** Flow cytometry analysis of infiltrated PD-1+ CTL in the 2208L tumors treated with vehicle or IACS-70654 for 18 days. For each group, five biological replicates were used. Two-tailed unpaired Student's *t* test was used. ns, p>0,05. Error bars represent SD. **C.** Changes in mouse weight of 2208L tumor-bearing mice of all treatment groups over days of treatment. Error bars represent SD. **D.** Tumor growth curves of 2208L tumors of all treatment groups.

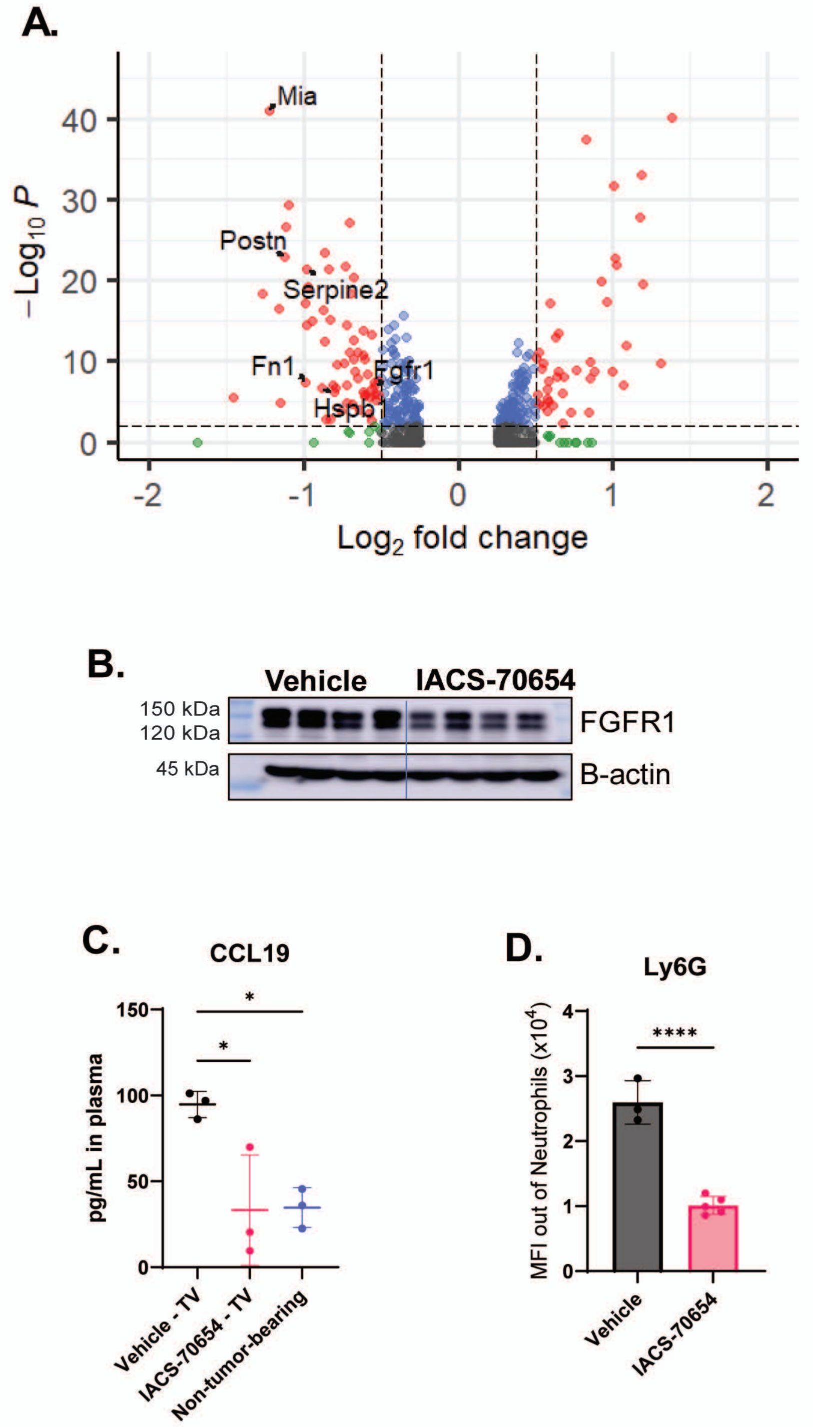
A.
Mia
Postn
Serpine2
Fn1
Fgfr1
Hspb1
−Log10 P
Log2 fold change
B.
Vehicle
IACS-70654
150 kDa
120 kDa
45 kDa
FGFR1
B-actin
C.
CCL19
pg/mL in plasma
Vehicle - TV
IACS-70654 - TV
Non-tumor-bearing
D.
Ly6G
MFI out of Neutrophils (x10^4)
Vehicle
IACS-70654

Supplemental Figure S7. Analysis of metastasis-related genes in 2208L tumors and 2208L lung metastases. **A.** Volcano plot showing –Log_{10} p-value versus Log_2 fold change in RNA expression in the tumor cells of 2208L tumors treated with IACS-70654 compared to those treated with vehicle. Genes that showed significant changes (Log_2 fold change > 0.5 or < -0.05 and adjusted p-value < 0.01) in expression are represented by red dots. The genes that are associated with tumor migration, invasion, and metastasis are labeled. **B.** Immunoblotting analysis of FGFR1 expression in 2208L tumors treated with vehicle or IACS-70654 for 7 days. For each treatment arm, four biological replicates were used. β-actin is used as the loading control. **C.** Quantification of CCL19 level in plasma from 2208L lung metastases-bearing mice treated with vehicle or IACS-70654 and non-tumor-bearing mice by the cytokine/chemokine array. Three biological replicates were used for each treatment arm. Ordinary one-way ANOVA and Tukey's multiple comparisons test were used. *, p<0.05. Error bars represent SD. **D.** Flow cytometry analyses showing median fluorescent intensity (MFI) of Ly6G in blood neutrophils of 2208L lung metastases-bearing mice treated with vehicle or IACS-70654. Two-tailed unpaired Student's *t* test was used. Error bars represent SD. ****, p<0.0001. Error bars represent SD.

References

1. Moon CI, et al. ClinicalOmicsDB: exploring molecular associations of oncology drug responses in clinical trials. *Nucleic Acids Res*. Jan 5 2024;52(D1):D1201-D1209.

	IACS-70654 (nM)
**CBP IC_{50}**	5.5
**BRD4 IC_{50}**	544

Supplemental Table S1. Specific binding of the CBP or BRD4 bromodomain by IACS-70654.

Target	Kd (nM)	Selectivity
**CBP**	0.096	1x
**P300**	0.15	1.6x
**BRD2(1)**<br>**BRD2(2)**	17<br>390	177x<br>4062x
**BRD3(1)**<br>**BRD3(2)**	45<br>89	469x<br>927x
**BRD4(1)**<br>**BRD4(2)**	24<br>210	250x<br>2188x
**BRDT(1)**<br>**BRDT(2)**	7.5<br>170	78x<br>1771x
**WDR9(2)**	16	167x

Supplemental Table S2. Kd and selectivity of IACS-70654 against 32 bromodomain proteins.

Supplemental Table S3. The list of genes in the MDSC gene signature published by Alshetaiwi et al (1).

Supplemental Table S4. Genes significantly enriched (adjusted p-value <0.01, log2 fold change >0.5) in integrated cluster 5 of TAMs (IFN-TAMs) from single-cell RNA sequencing (scRNA-seq) analysis of 2208L tumors treated with IACS-70654 for 7 days.

Supplemental Table S5. Significantly enriched GO pathways (gene count >5, adjusted p-value <0.05) in cluster 5 of TAMs (IFN-TAMs). Biological Process gene sets from the GO database were used.

Supplemental Table S6. Genes significantly enriched (adjusted p-value <0.01, log2 fold change >0.5) in integrated cluster 0 of TAMs from scRNA-seq analysis of 2208L tumors treated with IACS-70654 for 7 days.

Supplemental Table S7. Significantly enriched GO pathways (gene count >5, adjusted p-value <0.05) in integrated cluster 0 of TAMs. Biological Process gene sets from the GO database were used.

Supplemental Table S8. Genes significantly enriched (adjusted p-value <0.01, log2 fold change >0.5) in integrated cluster 1 of TAMs from scRNA-seq analysis of 2208L tumors treated with IACS-70654 for 7 days.

Supplemental Table S9. Genes significantly enriched (adjusted p-value <0.01, log2 fold change >0.5) in integrated cluster 2 of TAMs from scRNA-seq analysis of 2208L tumors treated with IACS-70654 for 7 days.

Supplemental Table S10. Genes significantly enriched (adjusted p-value <0.01, log2 fold change >0.5) in integrated cluster 3 of TAMs from scRNA-seq analysis of 2208L tumors treated with IACS-70654 for 7 days.

Supplemental Table S11. Genes significantly enriched (adjusted p-value <0.01, log2 fold change >0.5) in integrated cluster 4 of TAMs from scRNA-seq analysis of 2208L tumors treated with IACS-70654 for 7 days.

Supplemental Table S12. Genes significantly enriched (adjusted p-value <0.01, log2 fold change >0.5) in integrated cluster 6 of TAMs from scRNA-seq analysis of 2208L tumors treated with IACS-70654 for 7 days.

Supplemental Table S13. The list of genes in the IFN-stimulated neutrophil gene signature published by Benguigui et al (2).

Supplemental Table S14. Differentially expressed genes (DEGs) (adjusted p-value <0.01, |log2 fold change| > 0.5) in immature neutrophils of 2208L tumor-bearing mice treated with IACS-70654 versus vehicle.

Supplemental Table S15. Significantly upregulated GO pathways (gene count >5, adjusted p-value <0.05) in immature neutrophils of 2208L tumor-bearing mice treated with IACS-70654 versus vehicle. Biological Process gene sets from the GO database were used.

Supplemental Table S16. DEGs (adjusted p-value <0.01, |log2 fold change| > 0.5) in mature neutrophils of 2208L tumor-bearing mice treated with IACS-70654 versus vehicle.

Supplemental Table S17. Significantly upregulated and downregulated GO pathways (gene count >5, adjusted p-value <0.05) in mature neutrophils of 2208L tumor-bearing mice treated with IACS-70654 versus vehicle. Biological Process gene sets from the GO database were used.

Supplemental Table S18. DEGs (adjusted p-value <0.01, |log2 fold change| > 0.5) in IL-1β+ neutrophils of 2208L tumor-bearing mice treated with IACS-70654 versus vehicle.

Supplemental Table S19. Significantly upregulated and downregulated GO pathways (gene count >5, adjusted p-value <0.05) in IL-1β+ neutrophils of 2208L tumor-bearing mice treated with IACS-70654 versus vehicle. Biological Process gene sets from the GO database were used.

Supplemental Table S20. The list of genes in the neutrophil differentiation gene signature published by Weinreb et al (3).

Supplemental Table S21. DEGs (adjusted p-value <0.01, |log2 fold change| > 0.5) in cluter 4 HSPC (one of the CMP-1 clusters) of 2208L tumor-bearing mice treated with IACS-70654 versus vehicle.

Supplemental Table S22. Significantly downregulated GO pathways (gene count >5, adjusted p-value <0.05) in cluster 4 HSPC (one of the CMP-1 clusters) of 2208L tumor-bearing mice treated with IACS-70654 versus vehicle. Biological Process gene sets from the GO database were used.

Supplemental Table S23. DEGs (adjusted p-value <0.01, |log2 fold change| > 0.5) in CMP-1 of 2208L tumor-bearing mice treated with IACS-70654 versus vehicle.

Supplemental Table S24. DEGs (adjusted p-value <0.01, |log2 fold change| > 0.5) in GMP-1 of 2208L tumor-bearing mice treated with IACS-70654 versus vehicle.

Supplemental Table S25. DEGs (adjusted p-value <0.01, |log2 fold change| > 0.5) in HSCs of 2208L tumor-bearing mice treated with IACS-70654 versus vehicle.

Supplemental Table S26. DEGs (adjusted p-value <0.01, |log2 fold change| > 0.5) in MPPs of 2208L tumor-bearing mice treated with IACS-70654 versus vehicle.

Supplemental Table S27. DEGs (adjusted p-value <0.01, |log2 fold change| > 0.5) in tumor cells from 2208L tumors treated with IACS-70654 versus vehicle.

Supplemental Table S28. Significantly upregulated and downregulated GO pathways (gene count >5, adjusted p-value <0.05) in tumor cells from 2208L tumors treated with IACS-70654 versus vehicle. Biological Process gene sets from the GO database were used.

Supplemental Table S29. Raw data obtained from ClinicalOmicsDB containing gene expressions and pCR information from breast cancer patients treated with paclitaxel and pembrolizumab (4,5).

Supplemental Table S30. Flow cytometry antibodies.

References

1. Alshetaiwi H, et al. Defining the emergence of myeloid-derived suppressor cells in breast cancer using single-cell transcriptomics. *Sci Immunol*. Feb 21 2020;5(44)

2. Benguigui M, et al. Interferon-stimulated neutrophils as a predictor of immunotherapy response. *Cancer Cell*. Feb 12 2024;42(2):253-265 e12.

3. Weinreb C, et al. Lineage tracing on transcriptional landscapes links state to fate during differentiation. *Science*. Feb 14 2020;367(6479)

4. Wolf DM, et al. Redefining breast cancer subtypes to guide treatment prioritization and maximize response: Predictive biomarkers across 10 cancer therapies. *Cancer Cell*. Jun 13 2022;40(6):609-623 e6.

5. Moon CI, et al. ClinicalOmicsDB: exploring molecular associations of oncology drug responses in clinical trials. *Nucleic Acids Res*. Jan 5 2024;52(D1):D1201-D1209.
