# Supplementary material for: CREB-binding protein/P300 bromodomain inhibition reduces neutrophil accumulation and activates antitumor immunity in triple-negative breast cancer
Source: JCI Insight. 2024 Sep 17;9(20):e182621. doi: 10.1172/jci.insight.182621 (PMC11533985; doi:10.1172/jci.insight.182621)

Full edited blot for Figure S1D

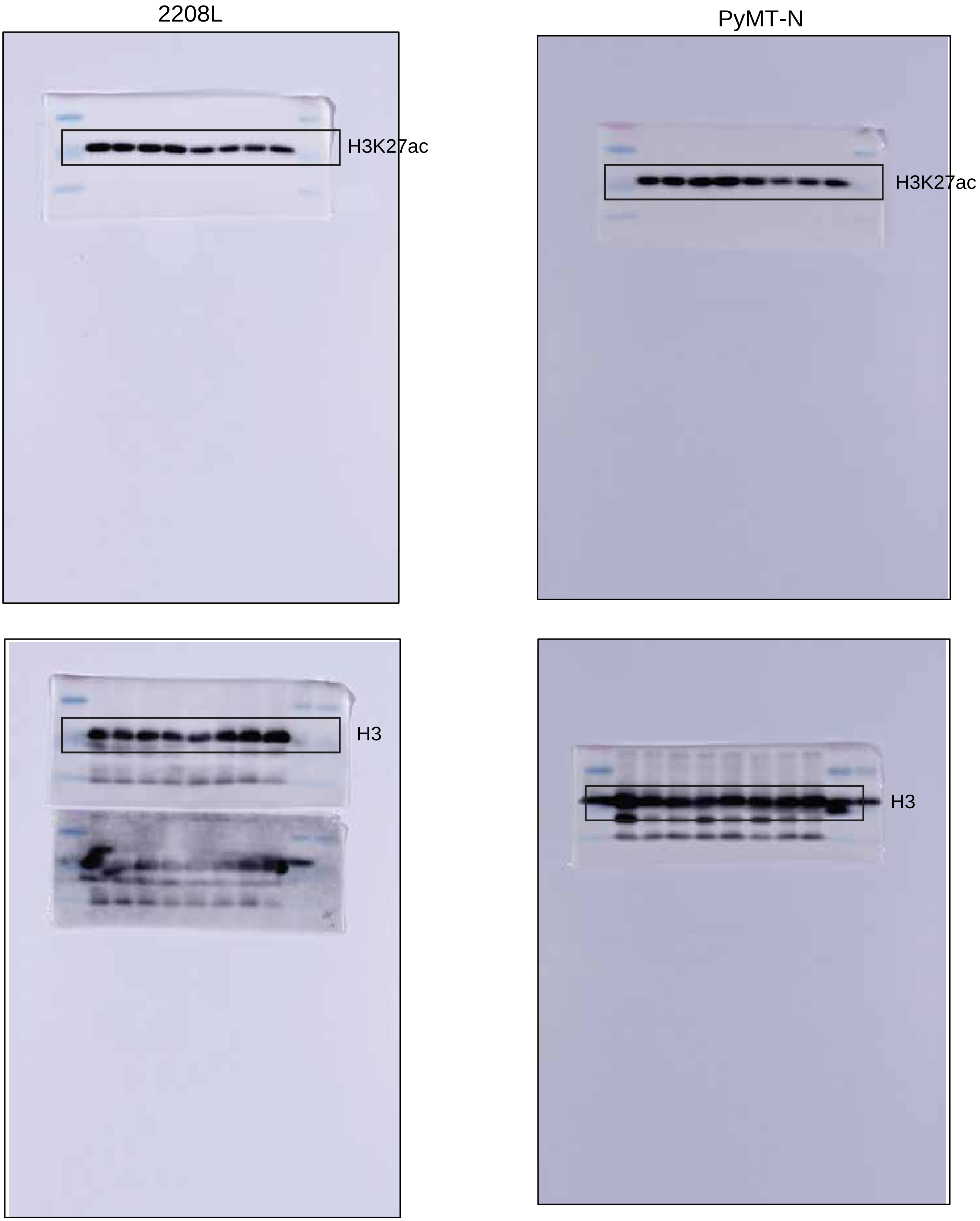

Full edited blot for Figure S1E

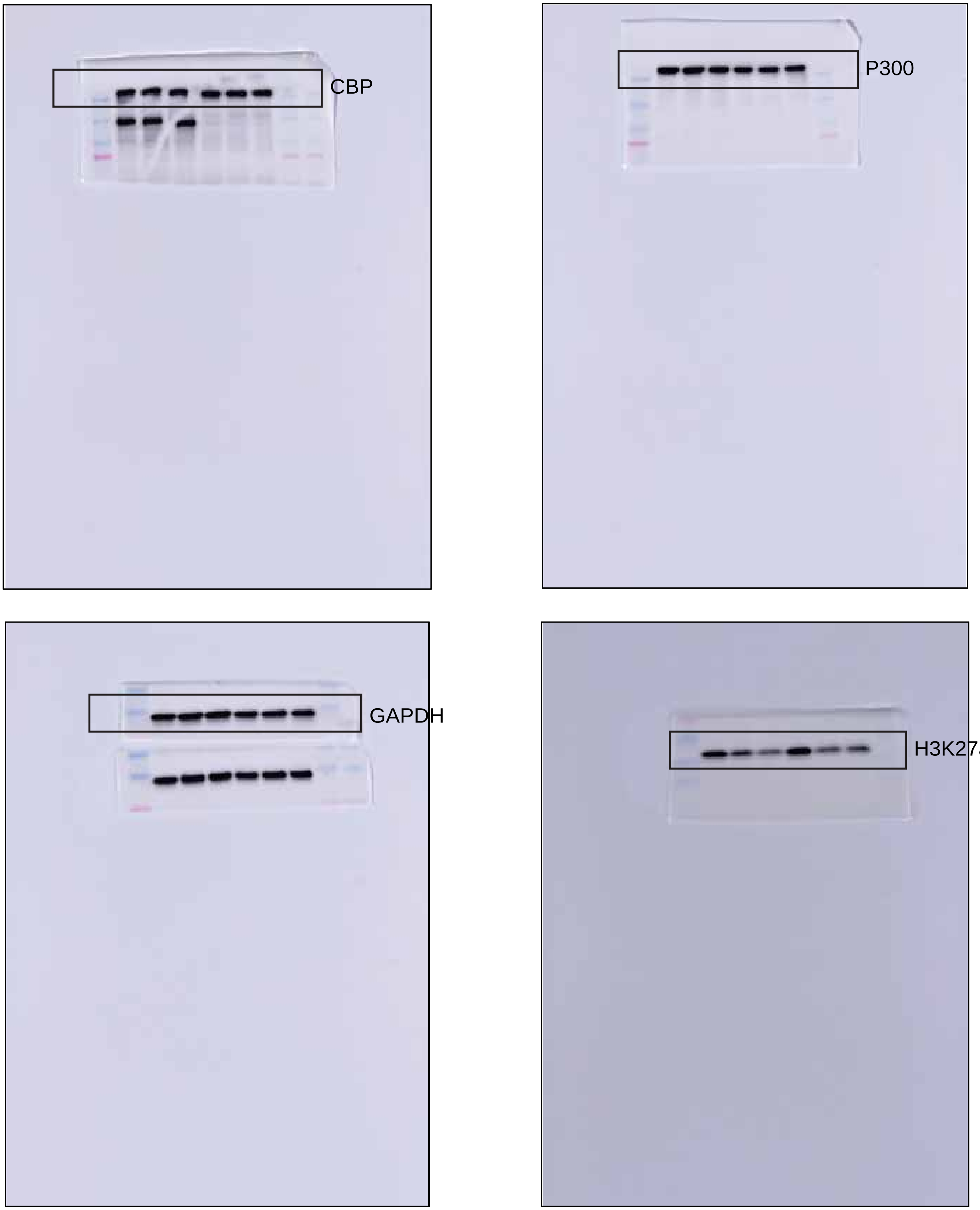

Full edited blot for Figure S1F

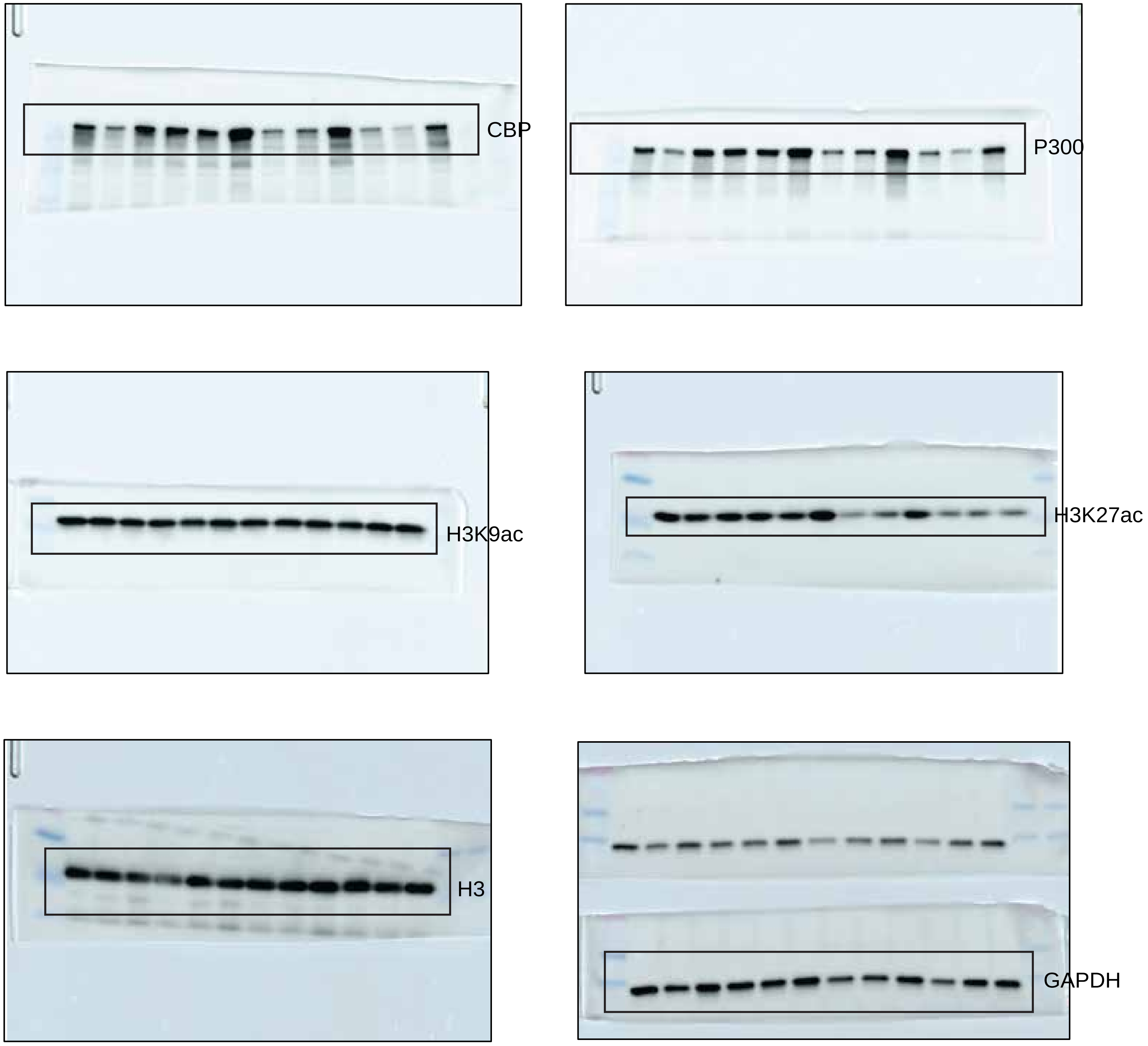

Full edited blot for Figure S7B

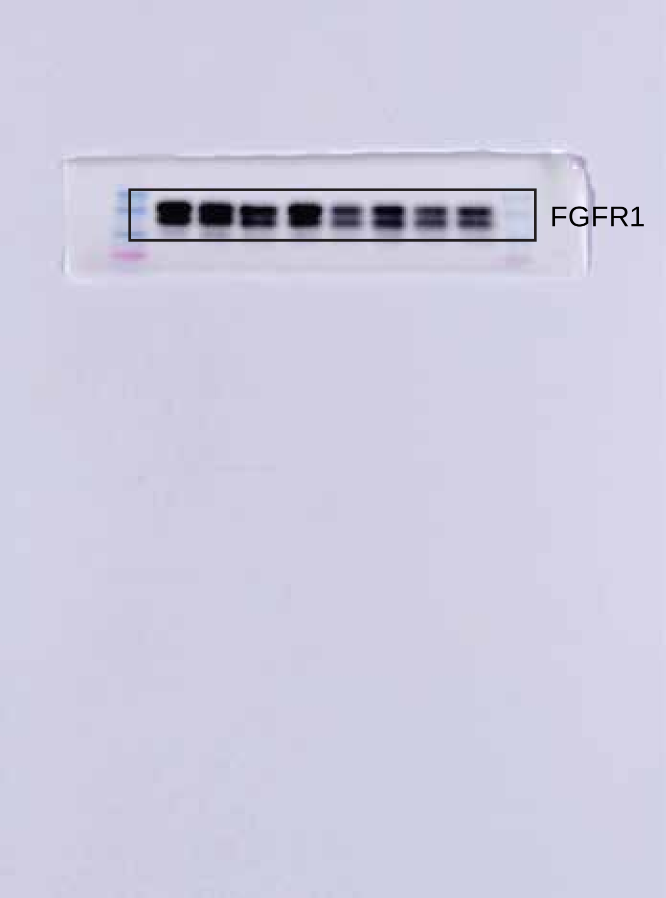

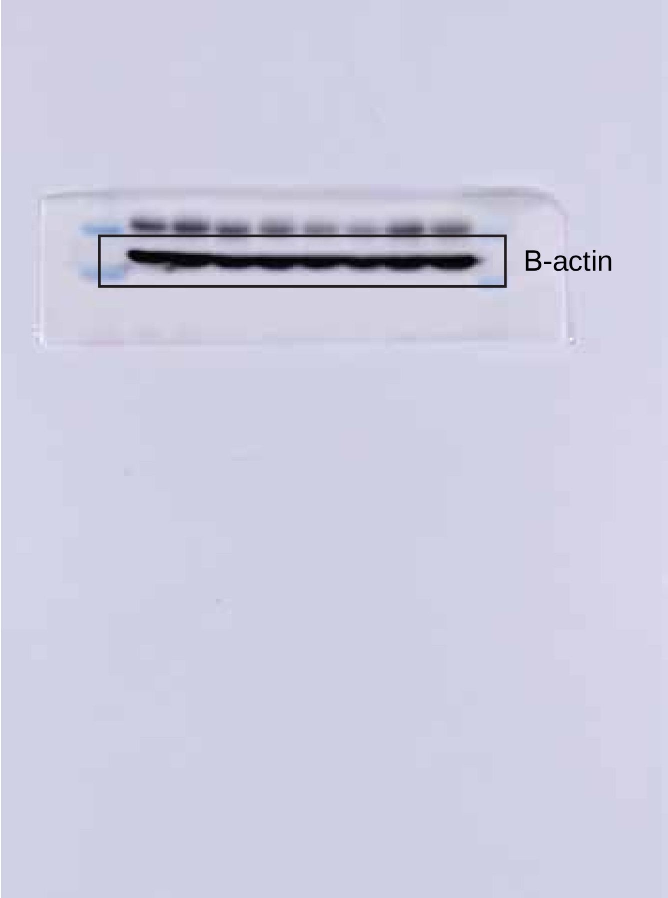

Supplement: Unedited blot and gel images [file jciinsight-9-182621-s009.pdf]
